# Solar UV-A radiation and blue light enhance tree leaf litter decomposition in a temperate forest

**DOI:** 10.1007/s00442-019-04478-x

**Published:** 2019-07-30

**Authors:** Marta Pieristè, Matthieu Chauvat, Titta K. Kotilainen, Alan G. Jones, Michaël Aubert, T. Matthew Robson, Estelle Forey

**Affiliations:** 1 Organismal and Evolutionary Biology (OEB), Viikki Plant Science Centre (ViPS), University of Helsinki, P.O. Box 65, Viikinkaari1, 00014 Helsinki, Finland; 2 Normandie Université, UNIROUEN, IRSTEA, ECODIV, FR Scale CNRS 3730, Rouen, France; 3 Earthwatch Institute, Mayfield House, 256 Banbury Road, Oxford, OX2 7DE UK; 4 Present Address: Natural Resources Institute Finland, Itäinen Pitkäkatu 4a, 20520 Turku, FI Finland; 5 Present Address: Forest Systems, Scion. 49 Sala Street, Private Bag 3020, Rotorua, 3046 New Zealand

**Keywords:** Photodegradation, C:N, Sunlight, Litter bags, Mass loss

## Abstract

Sunlight can accelerate the decomposition process through an ensemble of direct and indirect processes known as photodegradation. Although photodegradation is widely studied in arid environments, there have been few studies in temperate regions. This experiment investigated how exposure to solar radiation, and specifically UV-B, UV-A, and blue light, affects leaf litter decomposition under a temperate forest canopy in France. For this purpose, we employed custom-made litterbags built using filters that attenuated different regions of the solar spectrum. Litter mass loss and carbon to nitrogen (C:N) ratio of three species: European ash (*Fraxinus excelsior*), European beech (*Fagus sylvatica*) and pedunculate oak (*Quercus robur*), differing in their leaf traits and decomposition rate, were analysed over a period of 7–10 months. Over the entire period, the effect of treatments attenuating blue light and solar UV radiation on leaf litter decomposition was similar to that of our dark treatment, where litter lost 20–30% less mass and had a lower C:N ratio than under the full-spectrum treatment. Moreover, decomposition was affected more by the filter treatment than mesh size, which controlled access by mesofauna. The effect of filter treatment differed among the three species and appeared to depend on litter quality (and especially C:N), producing the greatest effect in recalcitrant litter (*F. sylvatica*). Even under the reduced irradiance found in the understorey of a temperate forest, UV radiation and blue light remain important in accelerating surface litter decomposition.

**Electronic supplementary material:**

The online version of this article (10.1007/s00442-019-04478-x) contains supplementary material, which is available to authorized users.

## Introduction

Photodegradation involves direct (photochemical mineralization) and indirect (photofacilitation) breakdown of organic matter mediated by sunlight which, alongside warm temperatures and high humidity, can accelerate the decomposition of plant litter (Brandt et al. 2007; Gallo et al. 2006, 2009; Almagro et al. 2015; Ma et al. 2017). Factors that enhance the exposure of plant litter to sunlight, such as changes to forest structure or phenology, modulate photodegradation and are an important environmental variable controlling decomposition rate in Mediterranean forests (Bravo-Oviedo et al. 2017; Gliksman et al. 2017). Decomposition rate partly governs nutrient cycling (Austin and Vivanco 2006) and successional processes in the plant and belowground communities (Fahey et al. 1998; Bardgett et al. 2005). Therefore, the interactions between the abiotic (sunlight, soil moisture, precipitation and temperature) and biotic drivers of decomposition have the potential to impact soil decomposer assemblages and plant functional composition in the understorey (Almagro et al. 2015). These interactions make it important to quantify the relative importance of photodegradation and contribution of different spectral regions to this process.

Short wavelengths of solar radiation carry high energy and can directly break down organic matter through photochemical mineralization (Gallo et al. 2006; Austin and Ballaré 2010). Until recently, most studies have considered only UV, or specifically UV-B (280–315 nm), radiation to be the main driver of photodegradation (reviewed by Song et al. 2013). However, recent studies have revealed that UV-A radiation (315–400 nm), blue (420–490 nm) and green (500–570 nm) regions of the spectrum (Sellaro et al. 2010) are also important in this process (Brandt et al. 2009; Austin and Ballaré 2010; Austin et al. 2016). The capacity of lignin, cellulose and hemicellulose to absorb UV radiation and blue and green light (Argyropoulos 2001; Austin and Ballaré 2010; Lin and King 2015) further suggests that these wavelengths are potentially involved in the photodegradation of litter. Solar radiation also affects decomposition rate through direct effects on both the activity (Duguay and Klironomos 2000) and community composition of decomposer organisms (Pancotto et al. 2003; Robson et al. 2005). Because these multiple environmental factors interact to produce complex effects, the relative contribution of photodegradation to decomposition is difficult to quantify.

Photodegradation has mainly been studied in habitats with a low-stature vegetation, such as grasslands or scrublands, where litter is exposed to near full sunlight all year round. In these environments, especially in arid and semiarid climates, photodegradation is particularly relevant (Gallo et al. 2009) and represents a key driver of the process of litter decomposition (Austin et al. 2016, but see King et al. 2012 and Song et al. 2013). Few studies have been undertaken in temperate environments and particularly in forest ecosystems (Messenger et al. 2012; Newsham et al. 2001), where decomposition is expected to be controlled by precipitation and temperature (Adair et al. 2008; Aerts 1997; Meentemeyer 1978). However, photodegradation can play a role in peat lands (Rutledge et al. 2010; Foereid et al. 2018), aquatic systems (Måns et al. 1998) and Arctic tundra (Cory et al. 2013) by interacting with microbial activity to produce a change in decomposition rate. This suggests that the ecological relevance of sunlight is not limited to dry environments receiving high irradiances of UV radiation but extends to Arctic and alpine environments (Foereid et al. 2011). There is a need to examine the extent to which photodegradation, and its interaction with decomposer organisms, contributes to decomposition in these environments to improve our estimation of how carbon cycling might be affected by climate change (Smith et al. 2012), which will expose litter to novel combinations of temperature, precipitation, day length and solar spectral irradiance. We aimed to test how the spectral composition of received solar radiation affects the decomposition of newly fallen leaf litter from three different tree species (*Fagus sylvatica* L.*, Quercus robur* L., and *Fraxinus excelsior* L.), on the floor of a temperate forest. We performed a litterbag experiment with five different sunlight attenuation filter treatments and two mesh treatments. We anticipated that the effect of photodegradation increases when the initial carbon to nitrogen (C:N) ratio is high (King et al. 2012) and expected that differences in initial litter quality according to species identity would lead to differing response in our sunlight attenuation treatments. Hence, we assessed litter decomposition of the three species over different time periods. We expected that UV radiation and blue light would enhance decomposition due to their capacity to break down organic material through photochemical mineralization (Gallo et al. 2009) and provide more nutrients for microbial activity as a result (photofacilitation, Austin et al. 2016). Consequently, we expected exposure to near-ambient UV radiation and blue light to lower the litter carbon content (Kotilainen et al. 2009; Almagro et al. 2017) and, therefore, the C:N ratio. The complexity of soil–decomposer assemblages is known to be important in the decomposition process (Hättenschwiler et al. 2005). Consequently, we expected that the exclusion of large decomposers (macrofauna and part of the mesofauna) from fine-mesh litterbags would interact with our filter treatments and produce different responses to the spectral regions of sunlight.

## Materials and methods

### Site description

The experiment was conducted in a mature beech forest (*Fagus sylvatica* L.) in Forêt Verte (49°31′12.6″N 1°07′00.7″E) close to Rouen University, France. The site has a relatively flat topography and the elevation is about 150 m a.s.l. The climate is “oceanic-temperate” with a mean annual air temperature of 10.5 °C and the total annual precipitation average of 851.7 mm, which is distributed relatively evenly over the year (ESM Fig. S1, climate data at the weather station “Rouen-Boos from 1981 to 2010”, data from website Infoclimat: http://www.infoclimat.fr).

**Fig. 1:**
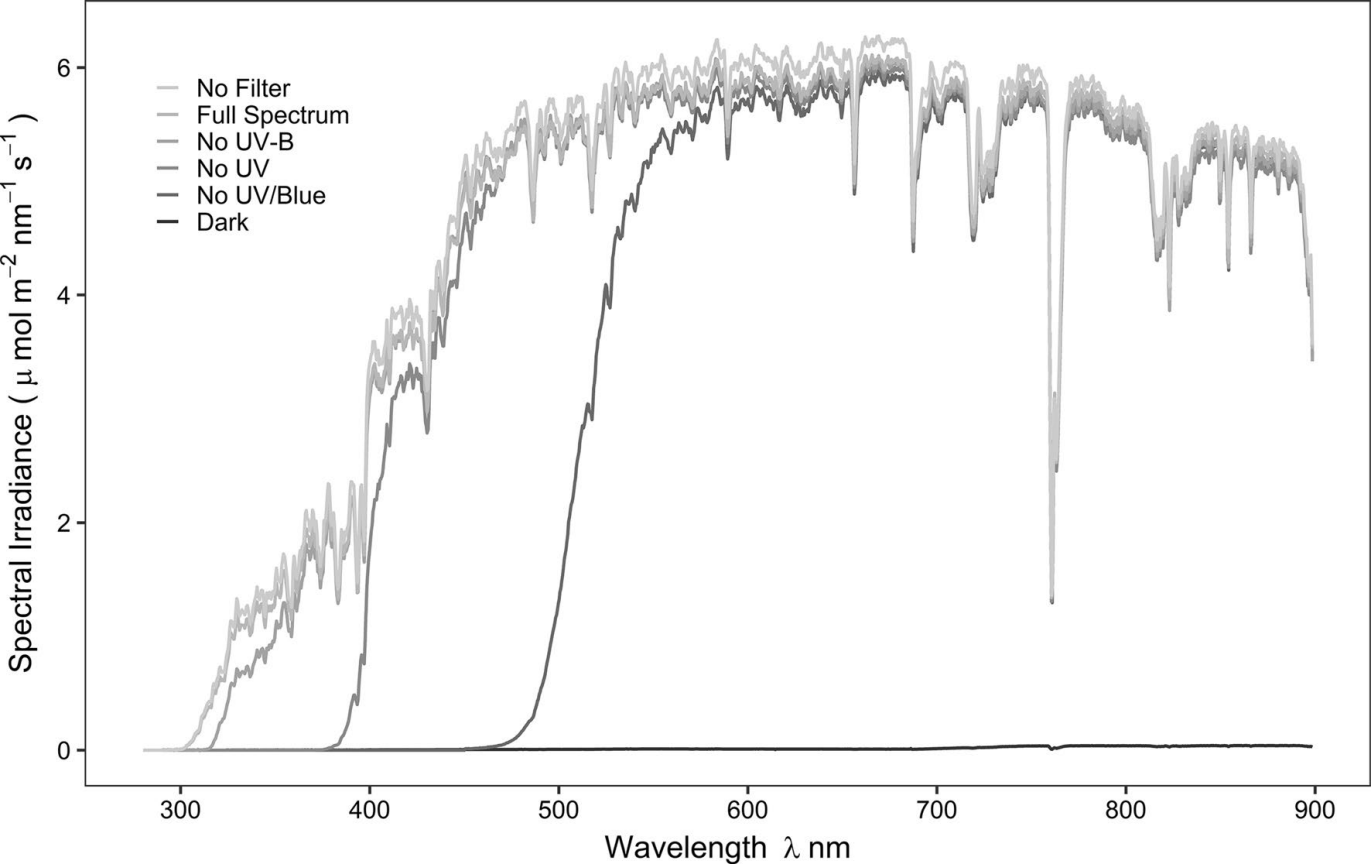
Measured spectral irradiance under the five filter treatments used in the experiment compared with ambient sunlight (no filter). Spectra were recorded with spectrometer at solar noon in Helsinki in July in an open area to measure the litterbags transmittance. Figure was produced using the photobiology packages in *R* (Aphalo 2015)

**Fig. 2:**
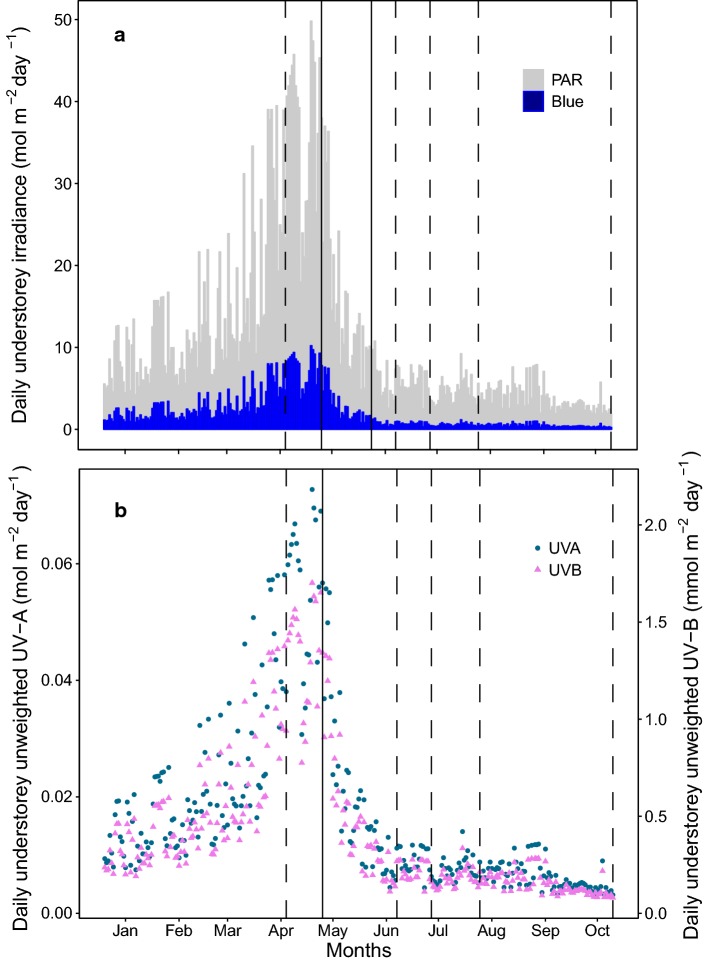
**a** Daily photosynthetically active radiation and blue light in the understorey. Time series of modelled PAR reconstructed using radiative transfer modelling of solar irradiance and the global light index (GLI) calculated from hemispherical photos taken at the site over the course of the experiment. Modelled data were cross-validated against a subset of daily measured PAR irradiance at the site from 25-05-2017 to 10-10-2017 (ESM Fig. S2). Vertical dashed lines show dates of litterbag collection, and solid line show the period of spring flush from bud burst to canopy closure from a visual assessment of the buds of canopy trees. **b** Daily estimated unweighted UV-A (filled circle) and UV-B (filled triangle) radiation in the understorey. Time series of solar irradiance were reconstructed using radiative transfer modelling, validated with above-canopy irradiance data provided by SoDa Helioclim-3, and gap light index calculated from hemispherical photos taken at the site over the course of the experiment. Vertical dashed line shows dates of litterbag collection, and solid lines show the period from bud burst to canopy closure from assessment of tree flush. (This figure is available in color in the online version of the journal)

Spectral irradiance was measured before (February 2017) and after (May 2017) canopy closure at five locations within the study site and compared with an open area nearby. Spectral irradiance was also measured inside the litterbags for each filter treatment to test filter transmittance (Fig. 1). Measurements were taken using an array spectroradiometer (Maya2000 Pro Ocean Optics, Dunedin, FL, USA; D7-H-SMA cosine diffuser, Bentham Instruments Ltd, Reading, UK) that had been calibrated within the previous 12 months for measurements spanning the regions of solar UV radiation and photosynthetically active radiation (PAR) (see Hartikainen et al. 2018 for details of the calibration, Aphalo et al. 2012, 2016). Hemispherical photos were taken on multiple occasions at the same five locations as the spectral irradiance measurements. To capture the different stages of canopy development, pictures were taken on 8th February 2017 when the canopy was dormant, during canopy flushing (once a week between 25th April 2017 and 30th May 2017) and after canopy closure (10th June 2017). These photos were used to characterize canopy cover by calculation of the global light index (GLI) and the leaf area index (LAI) with the software “Hemisfer” (Schleppi et al. 2007; Thimonier et al. 2010). The LAI was estimated to be 0.895 ± 0.012 during winter (Dec 2016–Apr 2017) corresponding to a GLI of 50.5%. On 24th May 2017, when canopy leaves were completely expanded, the LAI reached 2.930 ± 0.131 while the GLI dropped to 3.8%. A time series of modelled daily PAR (Fig. 2 and ESM Fig. S3) over the whole experimental period was reconstructed with a library of radiative transfer programs, libRadtran, version 2.0.1. (Emde et al. 2016). We used the radiative transfer equation solver DISORT for the simulations to produce spectra of 280–900 nm (based on Lindfors et al. 2009). Inputs to the model were column integrated water vapour data from AERONET (https://aeronet.gsfc.nasa.gov/cgi-bin/webtool_aod_v3?stage=2&place_code=10&region=Europe&state = France&submit = Get + AERONET + Sites), total ozone column data from the Aura Validation Data Center (AVDC) (https://avdc.gsfc.nasa.gov/pub/data/satellite/Aura/OMI/V03/L2OVP/OMUVB/) and surface type as defined by the International Geosphere Biosphere Programme (IGBP). Modelled above-canopy data were cross-validated against satellite-derived irradiance data provided by SoDa Helioclim-3 and against the spectral irradiance measured with the above-mentioned spectroradiometer. Modelled understorey data (Fig. 2 and ESM Table S12) were calculated by applying the GLI to the above-canopy modelled data (Canham 1988) and were cross-validated against a subset of daily PAR irradiance measured in the understorey on the forest floor, recorded continuously from 25th May to 10th Oct 2017 as 15-min averages with two calibrated quantum sensors (QSO-S, Decagon Devices, Pullman, Washington, USA) (ESM Fig S2). Estimates of received UV-A and UV-B radiation are given (Fig. 2, ESM Table S12) according to the spectral composition of modelled incident solar radiation without adjusting for the relative enrichment of UV radiation in shade which makes a minor contribution to the daily sum.

### Experimental design and litterbag design

We assigned litterbags to randomized locations within the study site (ESM Fig. S4). The experiment comprised 3 species of leaf litter × 5 filter treatments × 2 mesh sizes × 3 collection times × 5 replicates, giving a total number of 450 litterbags. The design of the litterbags for the experiment followed that described by Day et al. (2007). The dimensions of the litterbags were 150 × 150 mm, with the upper part made from a sheet of perforated film filter material and the bottom part made from a sterile Teflon mesh sheet of two different pore sizes: 0.1 mm allowing only microflora (fungi and bacteria) access to the litter, and 1 mm allowing microflora and part of the mesofauna (hereafter referred as mesofauna) to pass (ESM Figs. S5 and S6). The filter and the mesh sheet were not directly in contact but were held 8 mm apart by a frame made from plastic drinking straws (Ikea, Leiden, Netherlands), which helped to prevent contact between the leaves and the filter during decomposition. This separation was also important to prevent the build-up of condensation on the filter. Five different filter treatments were created (Fig. 1): a control treatment (full spectrum at near-ambient irradiance) of polyethene film (0.05 mm thick, 04 PE-LD; Etola, Jyväskylä, Finland) transmitting > 95% of incident PAR and UV radiation; no-UV-B treatment (attenuating UV-B radiation < 320 nm) using polyester (0.125 mm thick, Autostat CT5; Thermoplast, Helsinki, Finland); no-UV treatment using Rosco #226 (0.2 mm thick, West Lighting, Helsinki, Finland) attenuating UV radiation < 380 nm; no-UV/blue treatment using Rosco #312 Canary yellow (0.2 mm thick, West Lighting, Helsinki, Finland) attenuating UV radiation and blue light < 480 nm; and a dark treatment using polyethene film, solid white on the upper side and solid black on the lower side (0.15 mm thick, Casado Sarl, France), attenuating > 95% of PAR and UV radiation.

Litterbags were deployed on 20 Dec 2016, to coincide with the end of leaf fall and follow the natural timing of decomposition as faithfully as possible. They were pinned to the soil surface through a homogeneous thin layer of the previous years’ litter that remained in contact with the underside of the litterbags. Once a week, any debris that fell on the litterbags were removed, to ensure that they remained uncovered by other litter and unshaded by understorey plants. Air temperature and relative humidity (RH) inside a representative subsample of litterbags were continuously monitored with sensor ECH2O 5TM (Decagon Devices, Pullman, Washington, USA). The environment under the dark treatment was on average 0.4 °C (± 0.2) cooler (however, not statistically significant, ESM Table S14) and 1% (± 0.5) RH moister than the other treatments, while small-mesh-size (0.1 mm) bags were 0.8% (± 0.3) more moist than 1-mm mesh bags (ESM Tables S13 and S14).

### Litter material

Leaf litter was used from three widespread European tree species growing within the experimental area, selected according to their different litter quality: pedunculate oak (*Quercus robur* L.); European beech (*Fagus sylvatica* L.) and European ash (*Fraxinus excelsior* L.). The latter is known to produce labile litter with low lignin:N ratio of 13.6, able to decompose completely in 6–7 months (Melillo et al. 1982), oak litter represents intermediate-quality litter with a lignin:N ratio of 17.6 (Henneron et al. 2017) and beech produces more recalcitrant litter which decomposes over longer periods (up to 3 years) due to its higher lignin content (lignin:N ratio of 36.5: Trap et al. 2013). Fully senescent “sun” leaves at the point of abscission were sampled directly from trees on the southern edge of the stands. The point of abscission was determined as the moment when the leaf would detach without any effort in pulling it away from the branch. Leaves were collected from oak and ash trees in small stands near the University in Rouen (49°27′44.2″N 1°03′48.2″E), while the equivalent beech leaves were collected in the Forêt Verte (49°30′17.0″N 1°06′44.9″E) close to the study site. The petiole was removed from the leaves before they were weighed and scanned to obtain fresh weight (FW) and leaf area was calculated with the software WinFOLIA (Image analysis for plant science, Regent Instruments Inc., Nepean, Canada). Immediately after sampling, both leaf adaxial (upper) and abaxial (lower) epidermal flavonoid content and leaf chlorophyll content were optically assessed using a Dualex Scientific + (ForceA, Paris Orsay, France) device. This allowed us to verify that there were no initial differences in pigmentation or epidermal UV transmission among the leaves of each species (ESM Table S1). The leaves were then dried at 35 °C for 1 week and reweighed (dry weight: DW) before being placed in the litterbags (ESM Table S1). Entire leaves were placed inside litterbags with the adaxial leaf epidermis facing up in a single layer of non-overlapping litter (consisting of 2–5 leaves per litterbag, weighing 300–800 mg according to the species: EMS Fig. S5).

### Litter mass loss, and carbon and nitrogen content

Five replicate litterbags from each treatment combination were collected after 3, 5 and 7 months for ash litter, and 3, 6 and 10 months for oak and beech litter, as well as a zero-time sample from all species. After collection, litter was dried at 35 °C, cleaned with small brushes to eliminate any soil particles and worm casts present, and weighed on a precision balance (Entris 224i-1S, Sartorius Lab Instruments GmbH & Co. KG, Göttingen, Germany). The litter was then ground to a fine powder, and a quantity of 3–4 mg DW was used to determine the percentage of C and N content using a CN Soil Analyzer Flash 2000 (Thermo Scientific, Waltham, USA). Ash-free dry mass (AFDM) was determined by combustion of subsample of each replicate in a muffle oven at 550 °C for 12 h to allow quantification of mineral contamination, e.g. from worm casts and soil.

### Data analysis

Treatment effects for mass loss, C:N ratio, C and N content were tested for each species separately, due to their differing collection dates, using a three-way ANOVA including fixed experimental factors: filter, mesh size and time and respective interactions between them. The normal distribution of the residuals and homoscedasticity of variance were checked when performing the statistical analyses. Where a significant (*p* < 0.05) interaction was given by the ANOVA, the pairwise comparisons were tested (Function glht in Package Multicomp). Holm’s adjustment was used to account for multiple pairwise comparisons. All statistical analyses were performed in R version 3.3.3 (2017).

## Results

### Litter mass

**Fig. 3:**
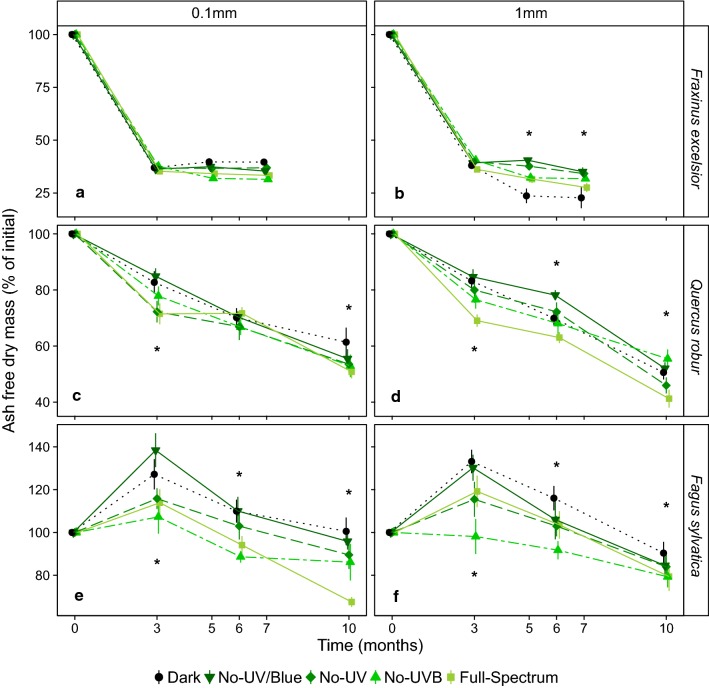
Remaining ash-free dry mass as a percentage of initial weight for each species litter: *F. excelsior* (**a**), (**b**); *Q. robur* (**c**),(**d**) and *F. Sylvatica* (**e**), (**f**), mesh size (0.1 mm and 1 mm) and filter treatment, over the 10 months of the experiment. Mean ± SE are shown (*n* = 5). *Dates with significant differences between the filter treatments. Pairwise comparisons were performed with the function glht in package Multicomp applying Holm’s adjustment. (This figure is available in color in the online version of the journal.)

The three species had different decomposition patterns confirming our initial hypothesis (Fig. 3). During its first 3 months, ash litter lost the largest proportion of its dry mass (60%) and by the time of its final collection (7 months) it had lost almost 70% of its initial dry mass. Oak litter decomposed much slower; only 50% mass was lost after 10 months, beech litter actually increased in mass during the first 3 months; this was particularly evident in the dark (+ 25%) and in the no-UV/blue (+ 40%) treatments (Fig. 3). This initial increase was followed by a decrease during the next 7 months, resulting in a 10–20% decrease from its original mass after 10 months (Fig. 3).

**Fig. 4:**
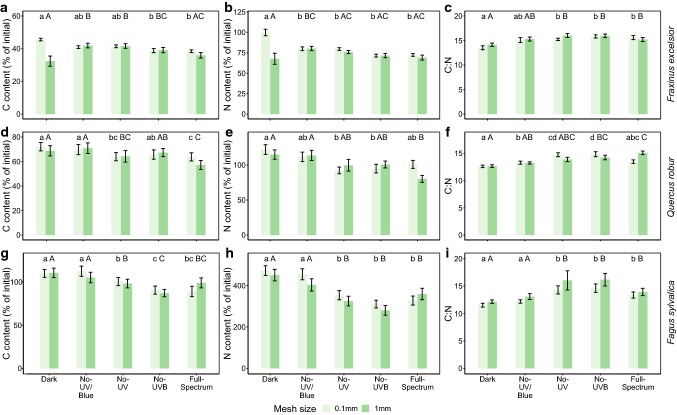
C content, N content and C:N ratio for each species litter: *F. excelsior* (**a**–**c**); *Q. robur* (**d**–**f**) and *F. Sylvatica* (**g**–**i**), mesh size (0.1 mm and 1 mm) and filter treatment. Means ± SE are shown (*n* = 15). Capital letters show significant differences between light treatments for mesh size = 1 mm. Lower case letters show significant differences between light treatments for mesh size = 0.1 mm. Pairwise comparisons were performed with the function glht in package Multicomp applying Holm’s adjustment. (This figure is available in color in the online version of the journal)

The effect of filter treatments on remaining mass of ash litter changed over time and according to the mesh size (Mesh × Filter × Time interaction: *p *= 0.032, Table 1, Figs. 3 and 4), suggesting a different effect of spectral composition on different groups of decomposers (micro- and part of the mesofauna). In both mesh sizes, there was no effect of filter treatment on remaining mass in the first 3 months (Figs. 3 and 4, ESM Table S2) suggesting photodegradation did not significantly contribute to the early phase of decomposition. After longer periods of decomposition, the effect of filter treatments differed only among the litter in 1 mm mesh-size litterbags. Significantly less mass remained under the dark filters (6%–10% less) than under the other filter treatments (ESM Table S3 and Fig. 4).

**Table 1:** ANOVA results for three fixed factors (Mesh: mesh size with two levels, Filter with five levels and Time with three levels) and their interactions on a single dependent variable: ash-free dry mass remaining for the three species’ litter

Factors	*d.f.*	SS	MS	*F*	*p*
**Ash (** ***Fraxinus excelsior*** **L.)**					
** Mesh**	**1**	**140**	**140.0**	**8.242**	**0.005**
**Filter**	**4**	**492**	**122.9**	**7.235**	**< 0.001**
**Time**	**2**	**612**	**306.0**	**18.019**	**< 0.001**
**Mesh × filter**	**4**	**795**	**198.7**	**11.701**	**< 0.001**
**Mesh × time**	**2**	**340**	**170.0**	**10.007**	**< 0.001**
Filter** × ** time	8	237	29.6	1.743	0.095
**Mesh × filter × time**	**8**	**299**	**37.3**	**2.198**	**0.032**
Residuals	120	2038	17.0		
**Oak (** ***Quercus robur*** **L.)**					
Mesh	1	61	60.7	1.158	0.284
**Filter**	**4**	**1786**	**446.5**	**8.517**	**< 0.001**
**Time**	**2**	**18,055**	**9027.5**	**172.210**	**< 0.001**
Mesh** × **filter	4	430	107.6	2.053	0.091
**Mesh × time**	**2**	**381**	**190.4**	**3.632**	**0.029**
Filter** × ** time	8	524	65.6	1.251	0.276
Mesh** × **filter** × ** time	8	419	52.4	1.001	0.439
Residuals	120	6291	52.4		
**Beech (** ***Fagus sylvatica*** **L.)**					
Mesh	1	31	31.4	0.163	0.687
**Filter**	**4**	**9881**	**2470.2**	**12.819**	**< 0.001**
**Time**	**2**	**29,176**	**14,588.1**	**75.705**	**< 0.001**
Mesh** × **filter	4	1190	297.5	1.544	0.1939
Mesh** × ** time	2	337	168.6	0.875	0.4195
Filter** × ** time	8	2323	290.4	1.507	0.162
Mesh** × **filter** × ** time	8	484	60.4	0.314	0.960
Residuals	120	23,124	192.7		

Degrees of freedom (*d.f.*), sum of squares (SS), mean square (MS), F statistic (F) and *p* value (*p*) are presented. Significant terms are shown in bold. Non-significant terms were retained since dropping them did not significantly affect the model

The effect of filter treatment on remaining mass of oak and beech litter depended on neither “time” nor “mesh size” (Mesh × Filter × Time interaction: *p *= 0.439 for oak litter and *p *= 0.960 for beech litter, Table 1, Fig. 3). For both oak and beech, more mass remained in the dark and no-UV/blue treatments than the full-spectrum treatment (ESM Table S4, Figs. 3 and 4), suggesting that the presence of blue light accelerated mass loss in litter of these two species. Beech litter actually gained mass during the first phase of decomposition, and 9.9% more litter remained in the no-UV treatment than the no-UV-B treatment (*p* = 0.031, ESM Table S4 and Fig. 4), i.e. the presence of UV-A radiation contributed to mass loss. There was no significant difference in mass loss from litter between the no-UV-B and full-spectrum treatments among any of the species (ESM Tables S3 and S4 and Figs. 3 and 4).

### Litter carbon and nitrogen content

The C content of the litter decreased over the decomposition period following a similar pattern to dry mass, while the N content increased in the early phases of decomposition (ESM Figs. S7 and S8); these relative changes in C and N resulted in a decrease in the C:N ratio over time (ESM Fig. S9). The effect of filter treatments on both C and N content in ash litter changed over time and according to the mesh size (Mesh × Filter × Time interaction: *p *= 0.014 and *p* = 0.048, respectively, Table 2, Fig. 4), suggesting again an effect of spectral composition on the interaction between different groups of decomposers. In both mesh sizes, there was no effect of light treatments on C and N content in the first 3 months (Fig. 4, ESM Tables S5 and S6). Following decomposition over longer time periods, the effect of filter treatments differed only for litter in litterbags with the 1 mm mesh size, with a significantly lower C content in the dark filters (− 6% to − 9% depending on the treatment) than the other filter treatments (ESM Table S7, Fig. 4). Considering N content, there was a significant effect of filter treatments only for litterbags with mesh size 0.1 mm. In these litterbags, the dark treatment produced litter with a higher N content (+ 19 to 27% depending on the treatment) than all other filter treatments (ESM Table S8, Fig. 4).

**Table 2:** ANOVA results of three fixed factors (Mesh: mesh size with two levels, Filter with five levels and Time with three levels) and their interactions on two single dependent variables: litter C content and litter N content for the three species’ litter

Species litter	Factors	Carbon content						Nitrogen content				
		*df*	SS	MS	*F*	*p*		*df*	SS	MS	*F*	*p*
**Ash (** ***Fraxinus excelsior*** **L.)**	**Mesh**	**1**	**304**	**304.2**	**15.999**	**< 0.001**		**1**	**2261.8**	**2261.8**	**18.746**	**< 0.001**
	**Filter**	**4**	**416**	**104.1**	**5.475**	**< 0.001**		**4**	**3755.0**	**938.5**	**7.780**	**< 0.001**
	**Time**	**2**	**1670**	**835.5**	**43.946**	**< 0.001**		**2**	**1036.2**	**518.1**	**4.294**	**0.016**
	**Mesh × filter**	**4**	**1054**	**263.7**	**13.871**	**< 0.001**		**4**	**5671.4**	**1417.9**	**11.751**	**< 0.001**
	**Mesh x Time**	**2**	**378**	**189.4**	**9.965**	**< 0.001**		**2**	**1962.0**	**980.9**	**8.130**	**< 0.001**
	**Filter x Time**	8	209	26.2	1.378	0.214		**8**	**2045.6**	**255.7**	**2.119**	**0.039**
	**Mesh x filter x time**	**8**	**384**	**48.1**	**2.528**	**0.014**		**8**	**1963.7**	**245.5**	**2.034**	**0.048**
	Residuals	120	2281	19.0				120	14,479.0	120.7		
**Oak (** ***Quercus robur*** **L.)**	Mesh	1	73	73.2	1.268	0.262		1	235	234.9	0.713	0.400
	**Filter**	**4**	**2159**	**539.8**	**9.355**	**< 0.001**		**4**	**16,969**	**4242.2**	**12.873**	**< 0.001**
	**Time**	**2**	**22,860**	**11,430.5**	**198.093**	**< 0.001**		**2**	**36,479**	**18,239.4**	**55.349**	**< 0.001**
	**Mesh x filter**	4	368	92.0	1.594	0.180		**4**	**4061**	**1015.3**	**3.081**	**0.019**
	**Mesh x time**	**2**	**434**	**217.3**	**3.767**	**0.026**		2	1557	778.7	2.363	0.099
	Filter x time	8	570	71.3	1.236	0.284		8	4365	545.6	1.656	0.116
	Mesh x filter x time	8	510	63.8	1.105	0.365		8	2690	336.3	1.021	0.424
	Residuals	120	694	57.7				120	39,544	329.5		
**Beech (** ***Fagus sylvatica*** **L.)**	Mesh	1	10	9.8	0.057	0.812		1	14,950	14,950	1.756	0.188
	**Filter**	**4**	**10,223**	**2555.7**	**14.809**	**< 0.001**		**4**	**566,543**	**141,636**	**16.635**	**< 0.001**
	**Time**	**2**	**35,906**	**17,953.0**	**104.032**	**< 0.001**		**2**	**93,088**	**46,544**	**5.467**	**0.005**
	Mesh x filter	4	1261	315.3	1.827	0.128		4	28,399	7100	0.834	0.506
	Mesh x time	2	171	85.5	0.496	0.611		2	10,348	5174	0.608	0.546
	Filter x time	8	1580	197.5	1.144	0.339		8	115,474	14,434	1.695	0.106
	Mesh x filter x time	8	565	70.6	0.409	0.9134		8	33,268	4158	0.488	0.862
	Residuals	120	20,709	172.6				120	1,021,697	8514		

Degrees of freedom (d.f.), sum of squares (SS), mean square (MS), F statistic (F) and *p* value (*p*) are presented. Significant terms are shown in bold. Non-significant terms were retained since dropping them did not significantly affect the model

For both oak and beech litter, there was no significant change in the effect of filter treatments on C and N content over time (Table 2, Fig. 4). For both species litter, there was no significant difference in C and N content between the dark and no-UV/blue treatments (Fig. 4, ESM Tables S9 and S10). These two treatments had the highest C content (Fig. 4, ESM Table S9), suggesting blue light stimulated C loss through photodegradation. Likewise, both oak and beech litter had the highest N contents in the dark and no-UV/blue treatments (Fig. 4, ESM Table S10), a sign of greater fungal colonization. For beech litter, the no-UV treatment had higher C content than the no-UV-B treatment (+ 9.9%, *p* = 0.031, Fig. 4 and ESM Table S9) implying that UV-A radiation was involved in promoting C loss. No significant difference in C content between the no-UV-B and full-spectrum treatments was found in any of the species’ litter (Fig. 4, ESM Tables S7, S8, S9, S10), suggesting that UV-B radiation was not involved in the process of C loss in our experiment.

## Discussion

The main findings of our experiment confirmed our expectations that litter decomposition would be significantly affected by solar radiation and its spectral attenuation in a temperate woodland, but that these responses would follow a different pattern according to initial litter quality and species identity. Oak and beech litter lost the greatest mass when exposed to the full-spectrum treatment, compared with treatments excluding UV radiation and both UV radiation and blue light, but this effect was not detected in ash litter. By the end of the experiment, litter exposed to the full-spectrum treatment lost between 20% (oak) and 30% (beech) more mass than litter in the dark treatment, and around 20% (both oak and beech) more mass than when both UV radiation and blue light were attenuated. These results develop further similar findings from past studies (Newsham et al. 2001; Messenger et al. 2012; King et al. 2012), showing that PAR/visible light interacts with UV-A and UV-B radiation to affect decomposition rates in temperate forests.

We are not able to infer the mechanism of response to blue light and UV-A radiation from our study. However, other experiments have found that lignin is able to absorb light in the blue and green range of the solar spectrum (Hon and Shiraishi 2001; Austin and Ballaré 2010), which may contribute to photochemical mineralization of lignin in the cell walls. For instance, Austin et al. (2016) found blue and green light to enhance litter decomposition via accelerated lignin breakdown in 23 temperate plant species. The increased bioavailability of cell-wall compounds through direct photodegradation may also prime this material for easier microbial colonization and breakdown by extracellular enzymes (Gallo et al. 2006; Baker and Allison 2015) via a so-called photofacilitation effect (Austin et al. 2016). In our experiment, exposure to blue light and UV-A radiation increased mass loss, while UV-B did not have any effect. Since microbial decomposition can be slowed by UV-B radiation (Lin et al. 2015, 2018; Wang et al. 2015), a trade-off may occur between the potential of UV-B radiation to break down organic matter and its capacity to decrease microbial activity and colonization (Verhoef et al. 2000). The importance of UV-B radiation in a forest understorey is also lessened because only approximately 2% of full sunlight is received during the period of canopy closure (Fig. 2).

The C:N ratio of litter from all three species decreased during the experiment, as a result of an overall increase in N content and a decrease in C content, which is consistent with other decomposition studies (Anderson 1973; Xuluc-Tolosa et al. 2003). This increase in N over time with declining mass has been observed in mesic environments, but it is not typical of arid environments where photodegradation plays a greater role (Parton et al. 2007). The litter C content in all those treatments receiving some portion of sunlight was lower than that of the dark treatment, which had the highest C content of all three species’ litter at the end of the experiment. These results corroborate an effect of solar radiation on C mobilization in a moist temperate forest which is in line with previous studies in arid, semiarid and subtropical biomes (Ma et al. 2017; Pan et al. 2015; Wang et al. 2017). Litter exposed to the full-spectrum treatment had a lower C content than litter receiving no-UV/blue light, in agreement with our hypothesis. This result suggests that blue light is involved in the breakdown of organic matter, as previously shown in a temperate grassland (Austin et al. 2016). Our results, together with previous studies, suggest that the PAR region of the spectrum is more important for photodegradation than the UV region in a temperate deciduous forest such as ours. This is not surprising given the far greater contribution of blue light than UV radiation to the received irradiance during dormancy in winter and before canopy closure in spring (Fig. 2, ESM Fig. S3, Grant et al. 2015; Hartikainen et al. 2018).

The filter treatments in our study had a smaller effect on ash litter than oak and beech litter. This reflects the importance of litter quality, and especially high initial C:N ratio, in determining the contribution of photodegradation to decomposition (reviewed by King et al. 2012), suggesting that microbial limitation due to low N content is likely to benefit most from photofacilitation. Similar trends occur in arid and semiarid environments (Gaxiola and Armesto 2015; Day et al. 2015) but are likely to be most relevant in moist environments where microbial decomposition dominates and the pool of fungal decomposers is far larger (Hodge et al. 2000). Furthermore, limiting the faunal groups able to colonize the litterbags (using a fine mesh) reduced the effect of light treatments on mass loss but increased this effect when considering litter N content. Soil fauna and microorganisms interact strongly during the decomposition process (Osler and Sommerkorn 2007); therefore, the interaction effect of our filter treatments with mesh size has implications for the relationships among these decomposers. This interaction, which is particularly evident in ash litter, suggests that functional groups of decomposers could have been differentially affected by spectral attenuation altering overall decomposition rates. However, further controlled experiments would be required to provide a mechanistic explanation for the patterns that we report here since our experiment did not consider the effect of macrofauna.

Beech litter gained mass during the first 3 months of decomposition; a similar increase in mass has been reported in studies addressing the first months of beech litter decomposition (Zeller et al. 2000; Idol et al. 2002; Brandstätter et al. 2013). Fungal colonization during the early phases of decomposition may account for this, as this is known to be particularly intense in beech litter compared to other species (Asplund et al. 2018) and fungal biomass can account for 23% of total detrital mass (Baldrian et al. 2013; Gulis et al. 2009; Gessner and Chauvet 2011). The strong correlation between change in mass and N content in beech litter over the first 3 months (*r*^2^ = 0.8–0.9 according to light treatment, ESM Fig. S10) suggests fungal colonization was the overwhelming process occurring during this period (Anderson 1973; Dickinson 1974; Zeller et al. 2000; d’Annunzio et al. 2008), presumably aided by the moist environment in our litterbags even with perforated filters. The higher N content of the litter in the absence of blue light and UV radiation is likely to be due to higher fungal biomass, because these wavelengths are known to inhibit the development of some fungi (De Lucca et al. 2012; Verhoef et al. 2000).

In our study of leaf litter decomposition in a moist temperate forest, UV-A radiation and blue light were found to have a more important role in photodegradation than UV-B radiation. This finding is consistent with other studies in similar climatic regions, in a dune grassland (Hoorens et al. 2004) and in a temperate woodland (Newsham et al. 2001), but differs from most arid (Day et al. 2007, 2015) and semiarid (Austin and Vivanco 2006) environments studied where UV-B radiation typically also increases mass loss. The relative importance of direct microbial inhibition by UV-B radiation reported in the literature vs. photochemical mineralization may provide an explanation for the different net effect of UV-B radiation on decomposition in a moist temperate ecosystem where biotic decomposition processes are more dominant than in drier ecosystems. The importance of photodegradation in arid and semiarid environments as a driver of carbon loss during decomposition is well known (Austin and Ballaré 2010; Austin et al. 2016); this study allows us to extend that finding to temperate forest environments, albeit acknowledging that this study focused on decomposition of the top layer of surface leaf litter and not buried material. Compared to grassland ecosystems, forest ecosystems have greater litter thickness and litter mass, and consequently a lower ratio of exposed litter. For instance, in the area where our study site is located, the typical litter layer thickness (OL) is about 1.5 (± 0.6) cm (Aubert et al. 2004), while leaf litter production is about 2.5 (± 0.5) t ha^−1^ yr^−1^ (Trap et al. 2011). While the effect of photodegradation will decrease with increasing litter layer thickness (Henry et al. 2008 and Mao et al. 2018), there remains potential for it to have a priming on surface litter, which would subsequently affect decomposition of covered litter due to photopriming (Lin et al. 2018). Photodegradation is able to mineralise up to 14% of NPP in arid systems and it is responsible for up to 23% of litter mass loss (King et al. 2012; Foereid et al. 2011); however, data are lacking from temperate forest environments. Knowing the role that photodegradation plays in decomposition is crucial to understanding its consequences for the global carbon cycle in forests, especially under a scenario of climate change. Within this framework, our results clearly suggest that parameterization of models designed to integrate photodegradation in the global carbon cycle should weight the wavelength regions of the solar spectrum differently, which is not yet the case (Foereid et al. 2011).

## Conclusion

This study found that even under the low solar irradiances in the understorey of a temperate forest, photodegradation, particularly by UV-A radiation and blue light, remains important in accelerating surface leaf litter decomposition (increasing mass loss by up to 30%). The extent of this effect is modulated by litter quality, which itself is known to depend on forest succession and light environment. This illustrates that sunlight is involved in mediating the rate of nutrient cycling in forest soils, not only through primary production but also through its effect on decomposition.

## Electronic supplementary material

Below is the link to the electronic supplementary material.

Supplementary material 1 (DOCX 2663 kb)
