# Supplementary material for: Solar UV-A radiation and blue light enhance tree leaf litter decomposition in a temperate forest
Source: Oecologia. 2019 Jul 30;191(1):191–203. doi: 10.1007/s00442-019-04478-x (PMC6732127; doi:10.1007/s00442-019-04478-x)
Supplement: Supplementary file 1 — Supplementary material 1 (DOCX 2663 kb) [file 442_2019_4478_MOESM1_ESM.docx]

**ELECTRONIC SUPPLEMENTARY MATERIAL**

##


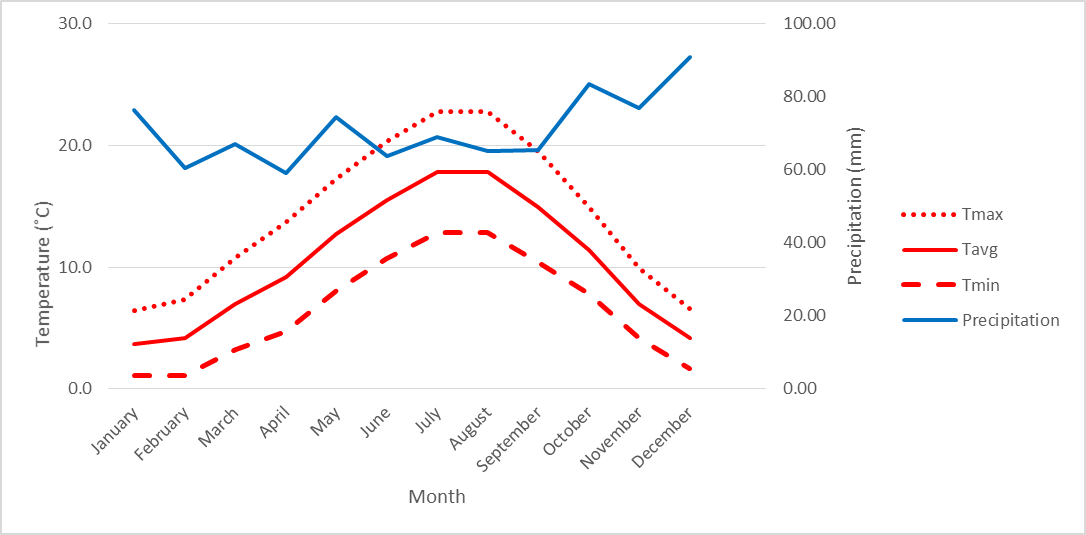


Figure S1 Climatic diagram of the study area (Rouen, France). Data from the weather station Rouen-Boos (49.38°N; 1.18°E) in Seine-Maritime, altitude 151 m.

Figure S2 Cross-validation of measured understorey PAR vs. a subset of modelled understorey PAR accounting for daily weather conditions for the period 25-05-2017 to 10-10-2017.


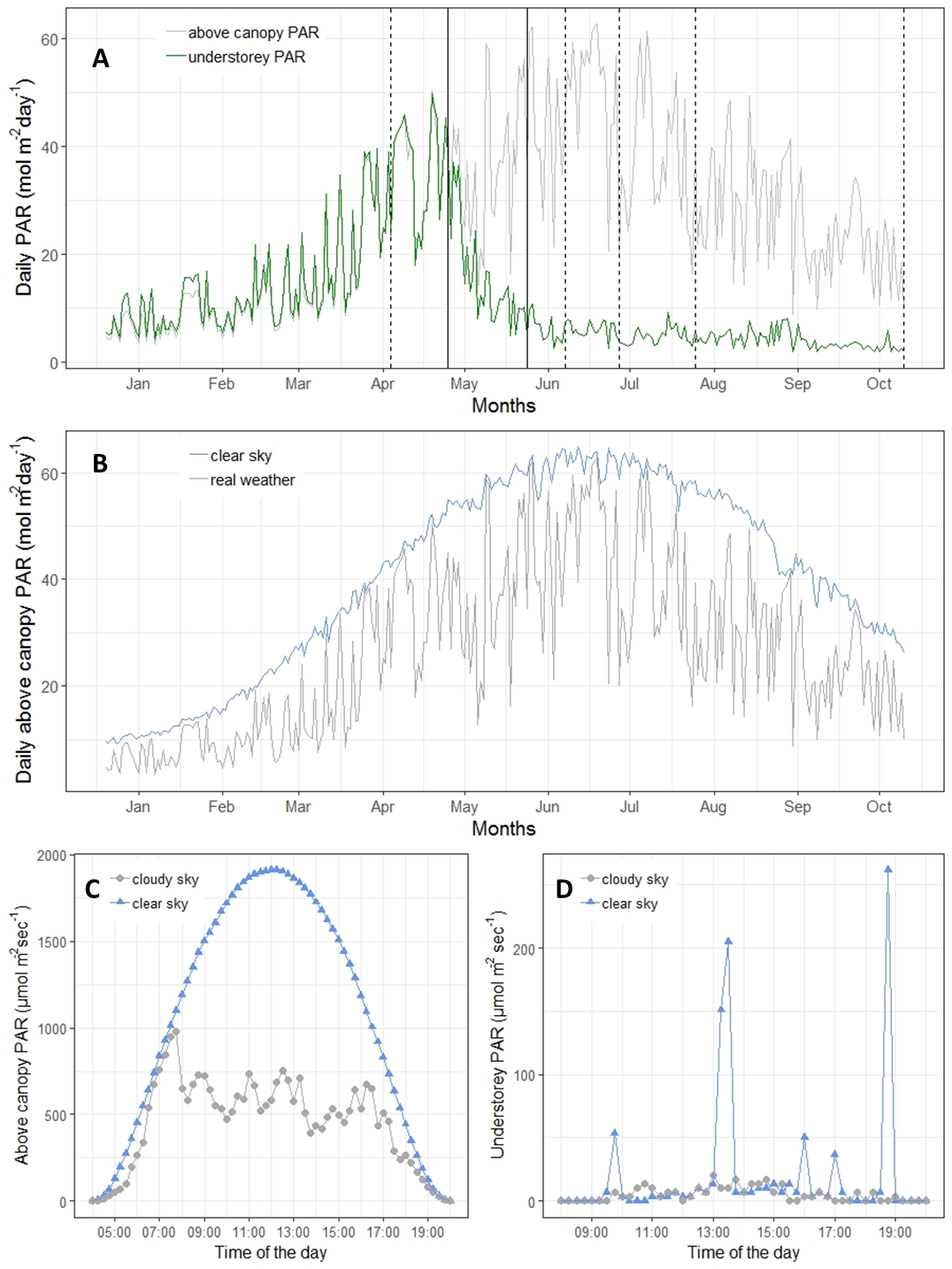


Figure S3 A) Daily photosynthetically active radiation (PAR) in the understorey (in green) and above the canopy (in grey). Time series of modelled PAR reconstructed using radiative transfer modelling of solar irradiance and global light index calculated from hemispherical photos taken at the site over the course of the experiment. Modelled data were cross-validated against a subset of daily measured PAR irradiance at the site from 25-05-2017 to 10-10-2017. Vertical dashed lines show dates of litterbag collection, and solid lines show the period of spring flush from bud burst to canopy closure from a visual assessment of the buds of canopy trees. B) Daily PAR above the canopy modelled for clear sky conditions (light blue) and accounting for actual weather conditions (grey). Modelled data validated with satellite data from SoDa Helioclim-3. C) Diurnal pattern of PAR above the canopy under clear and cloudy sky conditions at the field site at the end of May 2017. Modelled data validated with satellite data provided by SoDa Helioclim-3. D) Diurnal pattern of PAR in the understorey under clear and cloudy sky conditions at the end of May 2017. Data measured at the field site with two calibrated quantum sensors in parallel (QSO-S, Decagon Devices, Pullman, Washington, USA).

**
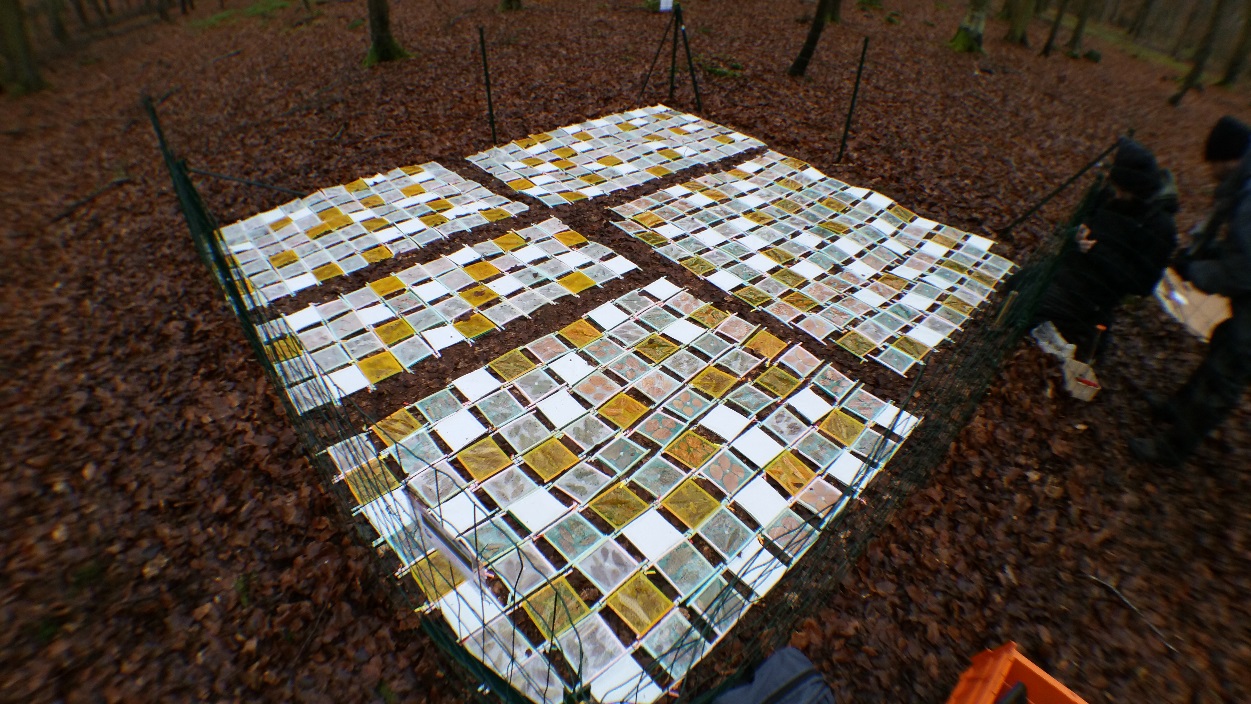
**

Figure S4 Picture showing the litterbags in the study site at the beginning of the experiment.

**
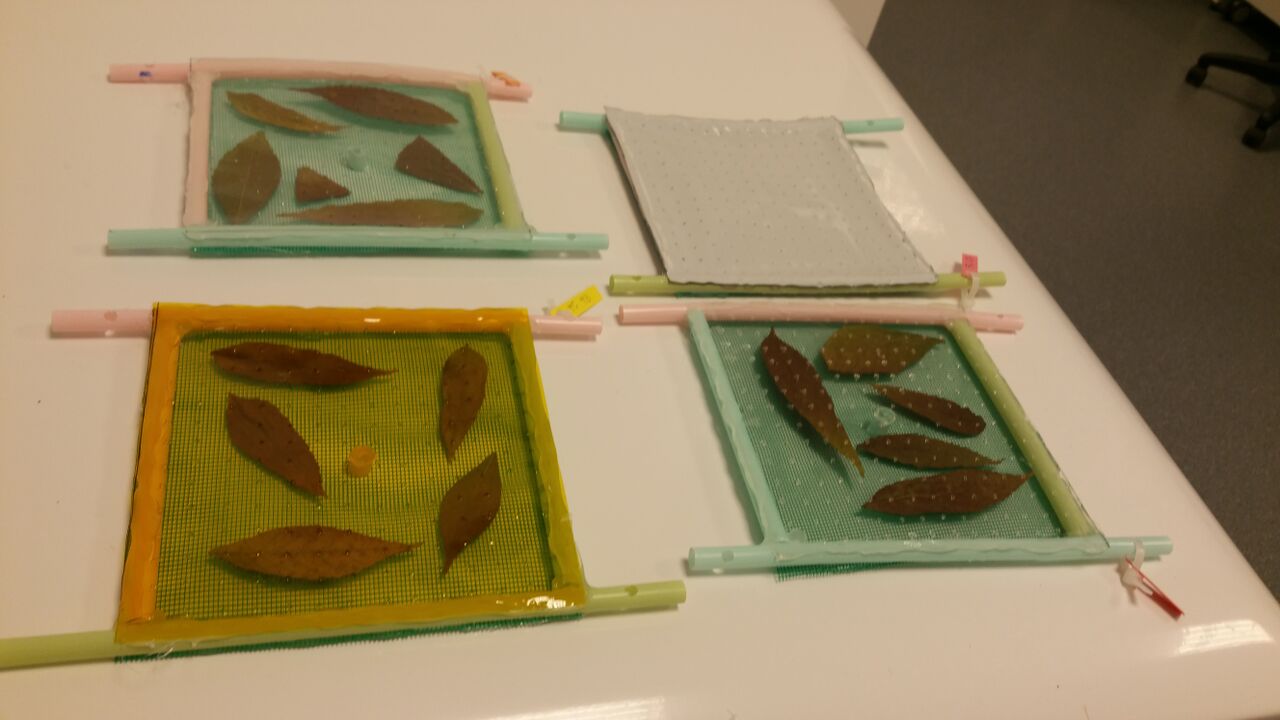
**

Figure S5 Picture showing some of the typical non-overlapping arrangements of leaves in the litterbags, as used in the experiment.


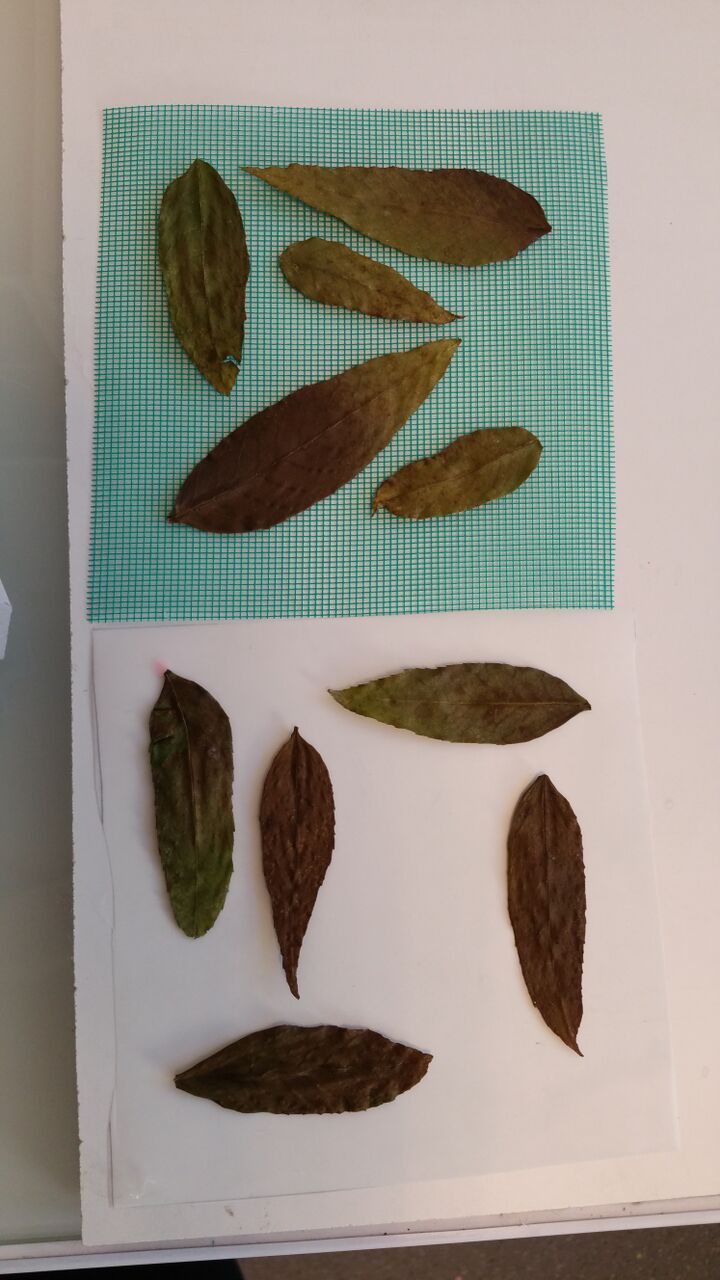


Figure S6 Picture showing an example of the mesh on the underside of the litterbags used in the experiment. Mesh size 0.1 mm on the left and mesh size 1 mm on the right.

| Species | Light treatment | FW (g) | DW (g) | Water Content (g) | LMA | Chl | Flav | C % | N % | C : N |
| --- | --- | --- | --- | --- | --- | --- | --- | --- | --- | --- |
| Ash | Dark | 1.60 ± 0.02 | 0.60 ± 0.01 | 0.37 ± 0.00 | 0.01 ± 0.00 | 18.10 ± 0.20 | 1.34 ± 0.01 | 39.45 ± 0.07 | 1.35 ± 0.01 | 29.18 ± 0.29 |
|  | Full-Spectrum | 1.50 ± 0.02 | 0.58 ± 0.01 | 0.39 ± 0.00 | 0.01 ± 0.00 | 17.41 ± 0.35 | 1.37 ± 0.01 | 39.45 ± 0.07 | 1.35 ± 0.01 | 29.18 ± 0.29 |
|  | No-UV | 1.45 ± 0.03 | 0.56 ± 0.01 | 0.38 ± 0.00 | 0.01 ± 0.00 | 16.47 ± 0.21 | 1.37 ± 0.01 | 39.45 ± 0.07 | 1.35 ± 0.01 | 29.18 ± 0.29 |
|  | No-UV/Blue | 1.51 ± 0.02 | 0.58 ± 0.01 | 0.38 ± 0.00 | 0.01 ± 0.00 | 17.21 ± 0.24 | 1.35 ± 0.01 | 39.45 ± 0.07 | 1.35 ± 0.01 | 29.18 ± 0.29 |
|  | No-UVB | 1.56 ± 0.01 | 0.59 ± 0.01 | 0.38 ± 0.00 | 0.01 ± 0.00 | 17.80 ± 0.23 | 1.38 ± 0.01 | 39.45 ± 0.07 | 1.35 ± 0.01 | 29.18 ± 0.29 |
| Beech | Dark | 0.90 ± 0.02 | 0.33 ± 0.01 | 0.36 ± 0.00 | 0.00 ± 0.00 | 6.32 ± 0.35 | 0.84 ± 0.02 | 43.67 ± 0.00 | 0.89 ± 0.03 | 48.94 ± 1.84 |
|  | Full-Spectrum | 0.96 ± 0.02 | 0.35 ± 0.01 | 0.37 ± 0.00 | 0.00 ± 0.00 | 6.72 ± 0.47 | 0.85 ± 0.02 | 43.67 ± 0.00 | 0.89 ± 0.03 | 48.94 ± 1.84 |
|  | No-UV | 0.97 ± 0.03 | 0.37 ± 0.01 | 0.39 ± 0.00 | 0.00 ± 0.00 | 6.90 ± 0.35 | 0.93 ± 0.02 | 43.67 ± 0.00 | 0.89 ± 0.03 | 48.94 ± 1.84 |
|  | No-UV/Blue | 0.90 ± 0.02 | 0.33 ± 0.01 | 0.37 ± 0.00 | 0.00 ± 0.00 | 6.23 ± 0.33 | 0.84 ± 0.02 | 43.67 ± 0.00 | 0.89 ± 0.03 | 48.94 ± 1.84 |
|  | No-UVB | 0.89 ± 0.02 | 0.32 ± 0.01 | 0.36 ± 0.00 | 0.00 ± 0.00 | 6.68 ± 0.35 | 0.84 ± 0.02 | 43.67 ± 0.00 | 0.89 ± 0.03 | 48.94 ± 1.84 |
| Oak | Dark | 1.94 ± 0.03 | 0.80 ± 0.01 | 0.41 ± 0.00 | 0.01 ± 0.00 | 11.81 ± 0.49 | 1.28 ± 0.01 | 43.79 ± 0.25 | 2.07 ± 0.09 | 21.23 ± 0.79 |
|  | Full-Spectrum | 1.81 ± 0.02 | 0.75 ± 0.01 | 0.41 ± 0.00 | 0.01 ± 0.00 | 12.11 ± 0.40 | 1.19 ± 0.01 | 43.79 ± 0.25 | 2.07 ± 0.09 | 21.23 ± 0.79 |
|  | No-UV | 1.89 ± 0.02 | 0.80 ± 0.01 | 0.42 ± 0.00 | 0.01 ± 0.00 | 31.24 ± 0.47 | 1.23 ± 0.01 | 43.79 ± 0.25 | 2.07 ± 0.09 | 21.23 ± 0.79 |
|  | No-UV/Blue | 2.04 ± 0.03 | 0.84 ± 0.01 | 0.41 ± 0.00 | 0.01 ± 0.00 | 12.25 ± 0.57 | 1.26 ± 0.01 | 43.79 ± 0.25 | 2.07 ± 0.09 | 21.23 ± 0.79 |
|  | No-UVB | 1.95 ± 0.02 | 0.81 ± 0.01 | 0.41 ± 0.00 | 0.01 ± 0.00 | 12.17 ± 0.55 | 1.29 ± 0.01 | 43.79 ± 0.25 | 2.07 ± 0.09 | 21.23 ± 0.79 |

Table S. 1 Initial litter quality, mean and standard errors are shown (n=5)

| **Mesh size: 0.1mm; Collection time: 3 months** | | | | |
| --- | --- | --- | --- | --- |
| **Filter** | **Estimate** | **SE** | **t-value** | **P value** |
| Dark - No-UV/Blue | 0.660 | 2.606 | 0.2532 | 1.000 |
| Dark - No-UV | 0.131 | 2.606 | 0.0502 | 1.000 |
| Dark - No-UVB | -0.636 | 2.606 | -0.2442 | 1.000 |
| Dark - Full-Spectrum | 1.723 | 2.606 | 0.6611 | 1.000 |
| No-UV/Blue - No-UV | -0.529 | 2.606 | -0.2030 | 1.000 |
| No-UV/Blue - No-UVB | -1.296 | 2.606 | -0.4973 | 1.000 |
| No-UV/Blue - Full-Spectrum | 1.063 | 2.606 | 0.4079 | 1.000 |
| No-UV - No-UVB | -0.767 | 2.606 | -0.2943 | 1.000 |
| No-UV - Full-Spectrum | 1.592 | 2.606 | 0.6109 | 1.000 |
| No-UVB - Full-Spectrum | 2.359 | 2.606 | 0.9052 | 1.000 |
| **Mesh size: 0.1mm; Collection time: 5 months** | | | | |
| **Filter** | **Estimate** | **SE** | **t-value** | **P value** |
| Dark - No-UV/Blue | 2.296 | 2.606 | 0.8810 | 1.000 |
| Dark - No-UV | 3.067 | 2.606 | 1.1765 | 1.000 |
| Dark - No-UVB | 7.806 | 2.606 | 2.9948 | 1.000 |
| Dark - Full-Spectrum | 5.569 | 2.606 | 2.1367 | 1.000 |
| No-UV/Blue - No-UV | 0.770 | 2.606 | 0.2956 | 1.000 |
| No-UV/Blue - No-UVB | 5.510 | 2.606 | 2.1139 | 1.000 |
| No-UV/Blue - Full-Spectrum | 3.273 | 2.606 | 1.2558 | 1.000 |
| No-UV - No-UVB | 4.739 | 2.606 | 1.8183 | 1.000 |
| No-UV - Full-Spectrum | 2.503 | 2.606 | 0.9602 | 1.000 |
| No-UVB - Full-Spectrum | -2.237 | 2.606 | -0.8581 | 1.000 |
| **Mesh size: 0.1mm; Collection time: 7 months** | | | | |
| **Filter** | **Estimate** | **SE** | **t-value** | **P value** |
| Dark - No-UV/Blue | 4.267 | 2.606 | 1.6371 | 1.000 |
| Dark - No-UV | 2.691 | 2.606 | 1.0326 | 1.000 |
| Dark - No-UVB | 8.179 | 2.606 | 3.1379 | 0.760 |
| Dark - Full-Spectrum | 6.312 | 2.606 | 2.4215 | 1.000 |
| No-UV/Blue - No-UV | -1.576 | 2.606 | -0.6045 | 1.000 |
| No-UV/Blue - No-UVB | 3.912 | 2.606 | 1.5009 | 1.000 |
| No-UV/Blue - Full-Spectrum | 2.045 | 2.606 | 0.7845 | 1.000 |
| No-UV - No-UVB | 5.488 | 2.606 | 2.1054 | 1.000 |
| No-UV - Full-Spectrum | 3.620 | 2.606 | 1.3890 | 1.000 |
| No-UVB - Full-Spectrum | -1.867 | 2.606 | -0.7164 | 1.000 |
| **Mesh size: 1mm; Collection time: 3 months** | | | | |
| **Filter** | **Estimate** | **SE** | **t-value** | **P value** |
| Dark - No-UV/Blue | -1.223 | 2.606 | -0.4691 | 1.000 |
| Dark - No-UV | -1.759 | 2.606 | -0.6748 | 1.000 |
| Dark - No-UVB | -2.471 | 2.606 | -0.9481 | 1.000 |
| Dark - Full-Spectrum | 1.880 | 2.606 | 0.7214 | 1.000 |
| No-UV/Blue - No-UV | -0.536 | 2.606 | -0.2056 | 1.000 |
| No-UV/Blue - No-UVB | -1.249 | 2.606 | -0.4790 | 1.000 |
| No-UV/Blue - Full-Spectrum | 3.103 | 2.606 | 1.1905 | 1.000 |
| No-UV - No-UVB | -0.713 | 2.606 | -0.2734 | 1.000 |
| No-UV - Full-Spectrum | 3.639 | 2.606 | 1.3962 | 1.000 |
| No-UVB - Full-Spectrum | 4.352 | 2.606 | 1.6695 | 1.000 |
| **Mesh size: 1mm; Collection time: 5 months** | | | | |
| **Filter** | **Estimate** | **SE** | **t-value** | **P value** |
| **Dark - No-UV/Blue** | **-16.860** | **2.606** | **-6.4686** | **< 0.001** |
| **Dark - No-UV** | **-14.020** | **2.606** | **-5.3790** | **< 0.001** |
| Dark - No-UVB | -8.565 | 2.606 | -3.2862 | 0.483 |
| Dark - Full-Spectrum | -7.924 | 2.606 | -3.0401 | 1.000 |
| No-UV/Blue - No-UV | 2.840 | 2.606 | 1.0896 | 1.000 |
| No-UV/Blue - No-UVB | 8.295 | 2.606 | 3.1824 | 0.670 |
| No-UV/Blue - Full-Spectrum | 8.936 | 2.606 | 3.4285 | 0.310 |
| No-UV - No-UVB | 5.455 | 2.606 | 2.0928 | 1.000 |
| No-UV - Full-Spectrum | 6.096 | 2.606 | 2.3389 | 1.000 |
| No-UVB - Full-Spectrum | 0.641 | 2.606 | 0.2461 | 1.000 |
| **Mesh size: 1mm; Collection time: 7 months** | | | | |
| **Filter** | **Estimate** | **SE** | **t-value** | **P value** |
| **Dark - No-UV/Blue** | **-12.365** | **2.606** | **-4.7439** | **0.002** |
| **Dark - No-UV** | **-11.445** | **2.606** | **-4.3909** | **0.010** |
| Dark - No-UVB | -9.048 | 2.606 | -3.4715 | 0.269 |
| Dark - Full-Spectrum | -4.871 | 2.606 | -1.8687 | 1.000 |
| No-UV/Blue - No-UV | 0.920 | 2.606 | 0.3531 | 1.000 |
| No-UV/Blue - No-UVB | 3.317 | 2.606 | 1.2724 | 1.000 |
| No-UV/Blue - Full-Spectrum | 7.494 | 2.606 | 2.8752 | 1.000 |
| No-UV - No-UVB | 2.396 | 2.606 | 0.9194 | 1.000 |
| No-UV - Full-Spectrum | 6.574 | 2.606 | 2.5221 | 1.000 |
| No-UVB - Full-Spectrum | 4.178 | 2.606 | 1.6028 | 1.000 |

Table S2 Pairwise comparisons for filter treatments on ash litter ash free dry mass (AFDM) according to mesh size and collection times: t- tests, with the Holm’s correction for multiple comparisons, were used to calculate the *P* values. Significant contrasts are shown in bold.

| **Mesh size: 0.1mm** | | | | |
| --- | --- | --- | --- | --- |
| **Filter** | **Estimate** | **SE** | **t-value** | **P value** |
| Dark - No-UV/Blue | 2.408 | 1.505 | 1.6000 | 1.000 |
| Dark - No-UV | 1.963 | 1.505 | 1.3044 | 1.000 |
| **Dark - No-UVB** | **5.116** | **1.505** | **3.3998** | **0.031** |
| Dark - Full-Spectrum | 4.535 | 1.505 | 3.0134 | 0.095 |
| No-UV/Blue - No-UV | -0.445 | 1.505 | -0.2956 | 1.000 |
| No-UV/Blue - No-UVB | 2.708 | 1.505 | 1.7998 | 1.000 |
| No-UV/Blue - Full-Spectrum | 2.127 | 1.505 | 1.4134 | 1.000 |
| No-UV - No-UVB | 3.153 | 1.505 | 2.0954 | 0.918 |
| No-UV - Full-Spectrum | 2.572 | 1.505 | 1.7090 | 1.000 |
| No-UVB - Full-Spectrum | -0.581 | 1.505 | -0.3864 | 1.000 |
| **Mesh size: 1mm** | | | | |
| **Filter** | **Estimate** | **SE** | **t-value** | **P value** |
| **Dark - No-UV/Blue** | **-10.149** | **1.505** | **-6.7444** | **< 0.001** |
| **Dark - No-UV** | **-9.075** | **1.505** | **-6.0302** | **< 0.001** |
| **Dark - No-UVB** | **-6.695** | **1.505** | **-4.4490** | **< 0.001** |
| Dark - Full-Spectrum | -3.638 | 1.505 | -2.4176 | 0.462 |
| No-UV/Blue - No-UV | 1.075 | 1.505 | 0.7142 | 1.000 |
| No-UV/Blue - No-UVB | 3.454 | 1.505 | 2.2954 | 0.586 |
| **No-UV/Blue - Full-Spectrum** | **6.511** | **1.505** | **4.3268** | **0.001** |
| No-UV - No-UVB | 2.380 | 1.505 | 1.5813 | 1.000 |
| **No-UV - Full-Spectrum** | **5.436** | **1.505** | **3.6126** | **0.016** |
| No-UVB - Full-Spectrum | 3.057 | 1.505 | 2.0314 | 1.000 |

Table S3 Pairwise comparisons for filter treatments on ash litter ash free dry mass (AFDM) according to mesh size: t- tests, with the Holm’s correction for multiple comparisons, were used to calculate the *P* values. Significant contrasts are shown in bold.

| **Oak (*Quercus robur* L.)** | | | | |
| --- | --- | --- | --- | --- |
| **Filter** | **Estimate** | **SE** | **t-value** | **P value** |
| Dark - No-UV/Blue | -1.267 | 1.883 | -0.6730 | 1.000 |
| Dark - No-UV | 4.527 | 1.883 | 2.4041 | 0.088 |
| Dark - No-UVB | 3.400 | 1.883 | 1.8057 | 0.220 |
| **Dark - Full-Spectrum** | **8.462** | **1.883** | **4.4933** | **< 0.001** |
| **No-UV/Blue - No-UV** | **5.795** | **1.883** | **3.0771** | **0.020** |
| No-UV/Blue - No-UVB | 4.668 | 1.883 | 2.4787 | 0.086 |
| **No-UV/Blue - Full-Spectrum** | **9.729** | **1.883** | **5.1663** | **< 0.001** |
| No-UV - No-UVB | -1.127 | 1.883 | -0.5984 | 1.000 |
| No-UV - Full-Spectrum | 3.934 | 1.883 | 2.0892 | 0.154 |
| No-UVB - Full-Spectrum | 5.061 | 1.883 | 2.6876 | 0.057 |
| **Beech (*Fagus sylvatica* L.)** | | | | |
| **Filter** | **Estimate** | **SE** | **t-value** | **P value** |
| Dark - No-UV/Blue | 2.028 | 3.580 | 0.5664 | 0.572 |
| **Dark - No-UV** | **11.079** | **3.580** | **3.0950** | **0.014** |
| **Dark - No-UVB** | **21.012** | **3.580** | **5.8694** | **< 0.001** |
| **Dark - Full-Spectrum** | **16.594** | **3.580** | **4.6353** | **< 0.001** |
| **No-UV/Blue - No-UV** | **9.052** | **3.580** | **2.5285** | **0.050** |
| **No-UV/Blue - No-UVB** | **18.984** | **3.580** | **5.3030** | **< 0.001** |
| **No-UV/Blue - Full-Spectrum** | **14.566** | **3.580** | **4.0689** | **< 0.001** |
| **No-UV - No-UVB** | **9.932** | **3.580** | **2.7745** | **0.031** |
| No-UV - Full-Spectrum | 5.514 | 3.580 | 1.5403 | 0.377 |
| No-UVB - Full-Spectrum | -4.418 | 3.580 | -1.2342 | 0.438 |

Table S4 Pairwise comparisons for filter treatments on beech and oak litter AFDM: t- tests, with the Holm’s correction for multiple comparisons, were used to calculate the *P* values. Significant contrasts are shown in bold.

| **Mesh size: 0.1mm; Collection time: 3 months** | | | | |
| --- | --- | --- | --- | --- |
| **Filter** | **Estimate** | **SE** | **t-value** | **P value** |
| Dark - No-UV/Blue | 1.188 | 2.758 | 0.4308 | 1.000 |
| Dark - No-UV | 1.933 | 2.758 | 0.7008 | 1.000 |
| Dark - No-UVB | 0.304 | 2.758 | 0.1101 | 1.000 |
| Dark - Full-Spectrum | 3.320 | 2.758 | 1.2039 | 1.000 |
| No-UV/Blue - No-UV | 0.745 | 2.758 | 0.2700 | 1.000 |
| No-UV/Blue - No-UVB | -0.884 | 2.758 | -0.3207 | 1.000 |
| No-UV/Blue - Full-Spectrum | 2.132 | 2.758 | 0.7730 | 1.000 |
| No-UV - No-UVB | -1.629 | 2.758 | -0.5907 | 1.000 |
| No-UV - Full-Spectrum | 1.387 | 2.758 | 0.5030 | 1.000 |
| No-UVB - Full-Spectrum | 3.016 | 2.758 | 1.0937 | 1.000 |
| **Mesh size: 0.1mm; Collection time: 5 months** | | | | |
| **Filter** | **Estimate** | **SE** | **t-value** | **P value** |
| Dark - No-UV/Blue | 5.298 | 2.758 | 1.9213 | 1.000 |
| Dark - No-UV | 5.915 | 2.758 | 2.1448 | 1.000 |
| Dark - No-UVB | 10.046 | 2.758 | 3.6431 | 0.144 |
| Dark - Full-Spectrum | 8.169 | 2.758 | 2.9623 | 1.000 |
| No-UV/Blue - No-UV | 0.616 | 2.758 | 0.2235 | 1.000 |
| No-UV/Blue - No-UVB | 4.748 | 2.758 | 1.7218 | 1.000 |
| No-UV/Blue - Full-Spectrum | 2.871 | 2.758 | 1.0411 | 1.000 |
| No-UV - No-UVB | 4.132 | 2.758 | 1.4983 | 1.000 |
| No-UV - Full-Spectrum | 2.254 | 2.758 | 0.8175 | 1.000 |
| No-UVB - Full-Spectrum | -1.877 | 2.758 | -0.6807 | 1.000 |
| **Mesh size: 0.1mm; Collection time: 7 months** | | | | |
| **Filter** | **Estimate** | **SE** | **t-value** | **P value** |
| Dark - No-UV/Blue | 7.364 | 2.758 | 2.6706 | 1.000 |
| Dark - No-UV | 4.453 | 2.758 | 1.6149 | 1.000 |
| Dark - No-UVB | 9.903 | 2.758 | 3.5913 | 0.169 |
| Dark - Full-Spectrum | 9.941 | 2.758 | 3.6049 | 0.163 |
| No-UV/Blue - No-UV | -2.911 | 2.758 | -1.0557 | 1.000 |
| No-UV/Blue - No-UVB | 2.539 | 2.758 | 0.9207 | 1.000 |
| No-UV/Blue - Full-Spectrum | 2.577 | 2.758 | 0.9343 | 1.000 |
| No-UV - No-UVB | 5.450 | 2.758 | 1.9764 | 1.000 |
| No-UV - Full-Spectrum | 5.488 | 2.758 | 1.9900 | 1.000 |
| No-UVB - Full-Spectrum | 0.038 | 2.758 | 0.0136 | 1.000 |
| **Mesh size: 1mm; Collection time: 3 months** | | | | |
| **Filter** | **Estimate** | **SE** | **t-value** | **P value** |
| Dark - No-UV/Blue | -0.978 | 2.758 | -0.3546 | 1.000 |
| Dark - No-UV | -1.371 | 2.758 | -0.4973 | 1.000 |
| Dark - No-UVB | -1.849 | 2.758 | -0.6705 | 1.000 |
| Dark - Full-Spectrum | 1.920 | 2.758 | 0.6962 | 1.000 |
| No-UV/Blue - No-UV | -0.393 | 2.758 | -0.1427 | 1.000 |
| No-UV/Blue - No-UVB | -0.871 | 2.758 | -0.3159 | 1.000 |
| No-UV/Blue - Full-Spectrum | 2.898 | 2.758 | 1.0508 | 1.000 |
| No-UV - No-UVB | -0.478 | 2.758 | -0.1732 | 1.000 |
| No-UV - Full-Spectrum | 3.291 | 2.758 | 1.1935 | 1.000 |
| No-UVB - Full-Spectrum | 3.769 | 2.758 | 1.3667 | 1.000 |
| **Mesh size: 1mm; Collection time: 5 months** | | | | |
| **Filter** | **Estimate** | **SE** | **t-value** | **P value** |
| **Dark - No-UV/Blue** | **-16.620** | **2.758** | **-6.0271** | **< 0.001** |
| **Dark - No-UV** | **-15.123** | **2.758** | **-5.4840** | **< 0.001** |
| Dark - No-UVB | -9.118 | 2.758 | -3.3065 | 0.415 |
| Dark - Full-Spectrum | -7.984 | 2.758 | -2.8954 | 1.000 |
| No-UV/Blue - No-UV | 1.498 | 2.758 | 0.5431 | 1.000 |
| No-UV/Blue - No-UVB | 7.502 | 2.758 | 2.7206 | 1.000 |
| No-UV/Blue - Full-Spectrum | 8.636 | 2.758 | 3.1318 | < 0.001 |
| No-UV - No-UVB | 6.005 | 2.758 | 2.1775 | 1.000 |
| No-UV - Full-Spectrum | 7.139 | 2.758 | 2.5887 | 1.000 |
| No-UVB - Full-Spectrum | 1.134 | 2.758 | 0.4112 | 1.000 |
| **Mesh size: 1mm; Collection time: 7 months** | | | | |
| **Filter** | **Estimate** | **SE** | **t-value** | **P value** |
| **Dark - No-UV/Blue** | **-11.025** | **2.758** | **-3.9980** | **0.042** |
| **Dark - No-UV** | **-11.294** | **2.758** | **-4.0957** | **0.030** |
| Dark - No-UVB | -9.233 | 2.758 | -3.3482 | 0.370 |
| Dark - Full-Spectrum | -4.558 | 2.758 | -1.6528 | 1.000 |
| No-UV/Blue - No-UV | -0.269 | 2.758 | -0.0977 | 1.000 |
| No-UV/Blue - No-UVB | 1.792 | 2.758 | 0.6497 | 1.000 |
| No-UV/Blue - Full-Spectrum | 6.467 | 2.758 | 2.3452 | 1.000 |
| No-UV - No-UVB | 2.061 | 2.758 | 0.7474 | 1.000 |
| No-UV - Full-Spectrum | 6.737 | 2.758 | 2.4429 | 1.000 |
| No-UVB - Full-Spectrum | 4.675 | 2.758 | 1.6955 | 1.000 |

Table S5 Pairwise comparisons for filter treatments on ash litter C content per mesh and collection times: t- tests, with the Holm’s correction for multiple comparisons, were used to calculate the *P* values. Significant contrasts are shown in bold.

| **Mesh size: 0.1mm; Collection time: 3 months** | | | | |
| --- | --- | --- | --- | --- |
| **Filter** | **Estimate** | **SE** | **t-value** | **P value** |
| Dark - No-UV/Blue | 17.976 | 6.947 | 2.5875 | 1.000 |
| Dark - No-UV | 13.483 | 6.947 | 1.9408 | 1.000 |
| Dark - No-UVB | 15.502 | 6.947 | 2.2315 | 1.000 |
| Dark - Full-Spectrum | 20.920 | 6.947 | 3.0114 | 1.000 |
| No-UV/Blue - No-UV | -4.493 | 6.947 | -0.6468 | 1.000 |
| No-UV/Blue - No-UVB | -2.474 | 6.947 | -0.3561 | 1.000 |
| No-UV/Blue - Full-Spectrum | 2.944 | 6.947 | 0.4238 | 1.000 |
| No-UV - No-UVB | 2.020 | 6.947 | 0.2907 | 1.000 |
| No-UV - Full-Spectrum | 7.438 | 6.947 | 1.0706 | 1.000 |
| No-UVB - Full-Spectrum | 5.418 | 6.947 | 0.7799 | 1.000 |
| **Mesh size: 0.1mm; Collection time: 5 months** | | | | |
| **Filter** | **Estimate** | **SE** | **t-value** | **P value** |
| Dark - No-UV/Blue | 13.560 | 6.947 | 1.9518 | 1.000 |
| Dark - No-UV | 22.146 | 6.947 | 3.1877 | 0.657 |
| **Dark - No-UVB** | **32.528** | **6.947** | **4.6822** | **0.003** |
| Dark - Full-Spectrum | 24.555 | 6.947 | 3.5345 | 0.221 |
| No-UV/Blue - No-UV | 8.586 | 6.947 | 1.2359 | 1.000 |
| No-UV/Blue - No-UVB | 18.968 | 6.947 | 2.7304 | 1.000 |
| No-UV/Blue - Full-Spectrum | 10.995 | 6.947 | 1.5827 | 1.000 |
| No-UV - No-UVB | 10.382 | 6.947 | 1.4945 | 1.000 |
| No-UV - Full-Spectrum | 2.409 | 6.947 | 0.3468 | 1.000 |
| No-UVB - Full-Spectrum | -7.973 | 6.947 | -1.1477 | 1.000 |
| **Mesh size: 0.1mm; Collection time: 7 months** | | | | |
| **Filter** | **Estimate** | **SE** | **t-value** | **P value** |
| **Dark - No-UV/Blue** | **28.025** | **6.947** | **4.0340** | **0.038** |
| Dark - No-UV | 25.150 | 6.947 | 3.6202 | 0.166 |
| **Dark - No-UVB** | **36.869** | **6.947** | **5.3071** | **< 0.001** |
| **Dark - Full-Spectrum** | **37.137** | **6.947** | **5.3456** | **< 0.001** |
| No-UV/Blue - No-UV | -2.874 | 6.947 | -0.4137 | 1.000 |
| No-UV/Blue - No-UVB | 8.845 | 6.947 | 1.2731 | 1.000 |
| No-UV/Blue - Full-Spectrum | 9.112 | 6.947 | 1.3116 | 1.000 |
| No-UV - No-UVB | 11.719 | 6.947 | 1.6869 | 1.000 |
| No-UV - Full-Spectrum | 11.986 | 6.947 | 1.7254 | 1.000 |
| No-UVB - Full-Spectrum | 0.268 | 6.947 | 0.0385 | 1.000 |
| **Mesh size: 1mm; Collection time: 3 months** | | | | |
| **Filter** | **Estimate** | **SE** | **t-value** | **P value** |
| Dark - No-UV/Blue | 12.422 | 6.947 | 1.7880 | 1.000 |
| Dark - No-UV | 10.072 | 6.947 | 1.4498 | 1.000 |
| Dark - No-UVB | 8.623 | 6.947 | 1.2412 | 1.000 |
| Dark - Full-Spectrum | 12.356 | 6.947 | 1.7786 | 1.000 |
| No-UV/Blue - No-UV | -2.350 | 6.947 | -0.3383 | 1.000 |
| No-UV/Blue - No-UVB | -3.799 | 6.947 | -0.5468 | 1.000 |
| No-UV/Blue - Full-Spectrum | -0.065 | 6.947 | -0.0094 | 1.000 |
| No-UV - No-UVB | -1.449 | 6.947 | -0.2085 | 1.000 |
| No-UV - Full-Spectrum | 2.285 | 6.947 | 0.3289 | 1.000 |
| No-UVB - Full-Spectrum | 3.733 | 6.947 | 0.5374 | 1.000 |
| **Mesh size: 1mm; Collection time: 5 months** | | | | |
| **Filter** | **Estimate** | **SE** | **t-value** | **P value** |
| **Dark - No-UV/Blue** | **-30.889** | **6.947** | **-4.4462** | **0.008** |
| Dark - No-UV | -17.603 | 6.947 | -2.5338 | 1.000 |
| Dark - No-UVB | -8.029 | 6.947 | -1.1557 | 1.000 |
| Dark - Full-Spectrum | -9.503 | 6.947 | -1.3678 | 1.000 |
| No-UV/Blue - No-UV | 13.286 | 6.947 | 1.9124 | 1.000 |
| No-UV/Blue - No-UVB | 22.860 | 6.947 | 3.2905 | 0.485 |
| No-UV/Blue - Full-Spectrum | 21.386 | 6.947 | 3.0784 | 0.913 |
| No-UV - No-UVB | 9.574 | 6.947 | 1.3781 | 1.000 |
| No-UV - Full-Spectrum | 8.100 | 6.947 | 1.1659 | 1.000 |
| No-UVB - Full-Spectrum | -1.474 | 6.947 | -0.2122 | 1.000 |
| **Mesh size: 1mm; Collection time: 7 months** | | | | |
| **Filter** | **Estimate** | **SE** | **t-value** | **P value** |
| Dark - No-UV/Blue | -19.189 | 6.947 | -2.7622 | 1.000 |
| Dark - No-UV | -16.977 | 6.947 | -2.4437 | 1.000 |
| Dark - No-UVB | -12.035 | 6.947 | -1.7323 | 1.000 |
| Dark - Full-Spectrum | -7.194 | 6.947 | -1.0355 | 1.000 |
| No-UV/Blue - No-UV | 2.212 | 6.947 | 0.3184 | 1.000 |
| No-UV/Blue - No-UVB | 7.155 | 6.947 | 1.0299 | 1.000 |
| No-UV/Blue - Full-Spectrum | 11.995 | 6.947 | 1.7266 | 1.000 |
| No-UV - No-UVB | 4.943 | 6.947 | 0.7114 | 1.000 |
| No-UV - Full-Spectrum | 9.783 | 6.947 | 1.4082 | 1.000 |
| No-UVB - Full-Spectrum | 4.841 | 6.947 | 0.6968 | 1.000 |

Table S6 Pairwise comparisons for filter treatments on ash litter N content per mesh and collection times: t- tests, with the Holm’s correction for multiple comparisons, were used to calculate the *P* values. Significant contrasts are shown in bold.

| **Mesh size: 0.1mm** | | | | |
| --- | --- | --- | --- | --- |
| **Filter** | **Estimate** | **SE** | **t-value** | **P value** |
| Dark - No-UV/Blue | 4.617 | 1.592 | 2.8999 | 0.129 |
| Dark - No-UV | 4.100 | 1.592 | 2.5753 | 0.314 |
| **Dark - No-UVB** | **6.751** | **1.592** | **4.2403** | **0.002** |
| **Dark - Full-Spectrum** | **7.143** | **1.592** | **4.4867** | **< 0.001** |
| No-UV/Blue - No-UV | -0.517 | 1.592 | -0.3245 | 1.000 |
| No-UV/Blue - No-UVB | 2.134 | 1.592 | 1.3405 | 1.000 |
| No-UV/Blue - Full-Spectrum | 2.526 | 1.592 | 1.5868 | 1.000 |
| No-UV - No-UVB | 2.651 | 1.592 | 1.6650 | 0.918 |
| No-UV - Full-Spectrum | 3.043 | 1.592 | 1.9113 | 1.000 |
| No-UVB - Full-Spectrum | 0.392 | 1.592 | 0.2463 | 1.000 |
| **Mesh size: 1mm** | | | | |
| **Filter** | **Estimate** | **SE** | **t-value** | **P value** |
| **Dark - No-UV/Blue** | **-9.541** | **1.592** | **-5.9928** | **< 0.001** |
| **Dark - No-UV** | **-9.263** | **1.592** | **-5.8180** | **< 0.001** |
| **Dark - No-UVB** | **-6.733** | **1.592** | **-4.2293** | **0.002** |
| Dark - Full-Spectrum | -3.541 | 1.592 | -2.2239 | 0.701 |
| No-UV/Blue - No-UV | 0.278 | 1.592 | 0.1748 | 1.000 |
| No-UV/Blue - No-UVB | 2.808 | 1.592 | 1.7635 | 1.000 |
| **No-UV/Blue - Full-Spectrum** | **6.000** | **1.592** | **3.7688** | **0.008** |
| No-UV - No-UVB | 2.529 | 1.592 | 1.5887 | 1.000 |
| **No-UV - Full-Spectrum** | **5.722** | **1.592** | **3.5940** | **0.015** |
| No-UVB - Full-Spectrum | 3.193 | 1.592 | 2.0053 | 1.000 |

Table S7 Pairwise comparisons for filter treatments on ash litter C content per mesh treatment: t- tests, with the Holm’s correction for multiple comparisons, were used to calculate the *P* values. Significant contrasts are shown in bold.

| **Mesh size: 0.1mm** | | | | |
| --- | --- | --- | --- | --- |
| **Filter** | **Estimate** | **SE** | **t-value** | **P value** |
| **Dark - No-UV/Blue** | **19.853** | **4.011** | **4.9498** | **< 0.001** |
| **Dark - No-UV** | **20.260** | **4.011** | **5.0511** | **< 0.001** |
| **Dark - No-UVB** | **28.300** | **4.011** | **7.0557** | **< 0.001** |
| **Dark - Full-Spectrum** | **27.537** | **4.011** | **6.8656** | **< 0.001** |
| No-UV/Blue - No-UV | 0.406 | 4.011 | 0.1013 | 1.000 |
| No-UV/Blue - No-UVB | 8.446 | 4.011 | 2.1058 | 1.000 |
| No-UV/Blue - Full-Spectrum | 7.684 | 4.011 | 1.9157 | 1.000 |
| No-UV - No-UVB | 8.040 | 4.011 | 2.0046 | 0.918 |
| No-UV - Full-Spectrum | 7.278 | 4.011 | 1.8145 | 1.000 |
| No-UVB - Full-Spectrum | -0.762 | 4.011 | -0.1901 | 1.000 |
| **Mesh size: 1mm** | | | | |
| **Filter** | **Estimate** | **SE** | **t-value** | **P value** |
| Dark - No-UV/Blue | -12.552 | 4.011 | -3.1294 | 0.079 |
| Dark - No-UV | -8.169 | 4.011 | -2.0368 | 1.000 |
| Dark - No-UVB | -3.813 | 4.011 | -0.9508 | 1.000 |
| Dark - Full-Spectrum | -1.447 | 4.011 | -0.3607 | 1.000 |
| No-UV/Blue - No-UV | 4.383 | 4.011 | 1.0927 | 1.000 |
| No-UV/Blue - No-UVB | 8.739 | 4.011 | 2.1787 | 0.939 |
| No-UV/Blue - Full-Spectrum | 11.105 | 4.011 | 2.7687 | 0.215 |
| No-UV - No-UVB | 4.356 | 4.011 | 1.0860 | 1.000 |
| No-UV - Full-Spectrum | 6.723 | 4.011 | 1.6761 | 1.000 |
| No-UVB - Full-Spectrum | 2.367 | 4.011 | 0.5900 | 1.000 |

Table S8 Pairwise comparisons for filter treatments on ash litter N content per mesh treatment: t- tests, with the Holm’s correction for multiple comparisons, were used to calculate the *P* values. Significant contrasts are shown in bold.

| **Oak (*Quercus robur* L.)** | | | | |
| --- | --- | --- | --- | --- |
| **Filter** | **Estimate** | **SE** | **t-value** | **P value** |
| Dark - No-UV/Blue | 0.082 | 1.997 | 0.0413 | 0.967 |
| **Dark - No-UV** | **6.305** | **1.997** | **3.1574** | **0.016** |
| Dark - No-UVB | 3.873 | 1.997 | 1.9397 | 0.272 |
| **Dark - Full-Spectrum** | **9.942** | **1.997** | **4.9791** | **< 0.001** |
| **No-UV/Blue - No-UV** | **6.222** | **1.997** | **3.1161** | **0.016** |
| No-UV/Blue - No-UVB | 3.791 | 1.997 | 1.8985 | 0.272 |
| **No-UV/Blue - Full-Spectrum** | **9.860** | **1.997** | **4.9378** | **< 0.001** |
| No-UV - No-UVB | -2.431 | 1.997 | -1.2177 | 0.451 |
| No-UV - Full-Spectrum | 3.637 | 1.997 | 1.8217 | 0.272 |
| **No-UVB - Full-Spectrum** | **6.069** | **1.997** | **3.0394** | **0.017** |
| **Beech (*Fagus sylvatica* L.)** | | | | |
| **Filter** | **Estimate** | **SE** | **t-value** | **P value** |
| Dark - No-UV/Blue | 1.432 | 3.365 | 0.4256 | 0.67 |
| **Dark - No-UV** | **10.860** | **3.365** | **3.2269** | **0.009** |
| **Dark - No-UVB** | **21.335** | **3.365** | **6.3393** | **< 0.001** |
| **Dark - Full-Spectrum** | **16.176** | **3.365** | **4.8064** | **< 0.001** |
| **No-UV/Blue - No-UV** | **9.428** | **3.365** | **2.8013** | **0.023** |
| **No-UV/Blue - No-UVB** | **19.902** | **3.365** | **5.9137** | **< 0.001** |
| **No-UV/Blue - Full-Spectrum** | **14.743** | **3.365** | **4.3808** | **< 0.001** |
| **No-UV - No-UVB** | **10.475** | **3.365** | **3.1124** | **0.011** |
| No-UV - Full-Spectrum | 5.316 | 3.365 | 1.5795 | 0.349308 |
| No-UVB - Full-Spectrum | -5.159 | 3.365 | -1.5329 | 0.349308 |

Table S9 Pairwise comparisons for filter treatments on beech and oak litter C content: t- tests, with the Holm’s correction for multiple comparisons, were used to calculate the *P* values. Significant contrasts are shown in bold.

| **Oak (*Quercus robur* L.)** | | | | |
| --- | --- | --- | --- | --- |
| **Filter** | **Estimate** | **SE** | **t-value** | **P value** |
| Dark - No-UV/Blue | 5.823 | 4.779 | 1.2184 | 0.676 |
| **Dark - No-UV** | **22.564** | **4.779** | **4.7210** | **< 0.001** |
| **Dark - No-UVB** | **20.736** | **4.779** | **4.3386** | **< 0.001** |
| **Dark - Full-Spectrum** | **27.929** | **4.779** | **5.8435** | **< 0.001** |
| **No-UV/Blue - No-UV** | **16.741** | **4.779** | **3.5026** | **0.004** |
| **No-UV/Blue - No-UVB** | **14.913** | **4.779** | **3.1202** | **0.011** |
| **No-UV/Blue - Full-Spectrum** | **22.105** | **4.779** | **4.6251** | **< 0.001** |
| No-UV - No-UVB | -1.828 | 4.779 | -0.3824 | 0.703 |
| No-UV - Full-Spectrum | 5.365 | 4.779 | 1.1225 | 0.676 |
| No-UVB - Full-Spectrum | 7.192 | 4.779 | 1.5049 | 0.539 |
| **Beech (*Fagus sylvatica* L.)** | | | | |
| **Filter** | **Estimate** | **SE** | **t-value** | **P value** |
| Dark - No-UV/Blue | 32.311 | 23.889 | 1.3525 | 0.357 |
| **Dark - No-UV** | **121.617** | **23.889** | **5.0909** | **< 0.001** |
| **Dark - No-UVB** | **165.686** | **23.889** | **6.9356** | **< 0.001** |
| **Dark - Full-Spectrum** | **117.593** | **23.889** | **4.9224** | **< 0.001** |
| **No-UV/Blue - No-UV** | **89.307** | **23.889** | **3.7384** | **0.002** |
| **No-UV/Blue - No-UVB** | **133.376** | **23.889** | **5.5831** | **< 0.001** |
| **No-UV/Blue - Full-Spectrum** | **85.282** | **23.889** | **3.5699** | **0.002** |
| No-UV - No-UVB | 44.069 | 23.889 | 1.8447 | 0.201 |
| No-UV - Full-Spectrum | -4.025 | 23.889 | -0.1685 | 0.866 |
| No-UVB - Full-Spectrum | -48.094 | 23.889 | -2.0132 | 0.184 |

Table S10 Pairwise comparisons for filter treatments on beech and oak litter N content: t- tests, with the Holm’s correction for multiple comparisons, were used to calculate the *P* values. Significant contrasts are shown in bold.

| **Ash (*Fraxinus excelsior* L.)** | | | | | | | | | | | | | | |  |
| --- | --- | --- | --- | --- | --- | --- | --- | --- | --- | --- | --- | --- | --- | --- | --- |
| **Factors** | **d.f.** | | | **SS** | | | | **MS** | | **F** | ***p*** | | | |  |
| Mesh | 1 | | | 2.857 | | | | 2.857 | | 1.7595 | 0.187 | | | |  |
| **Filter** | **4** | | | **77.735** | | | | **19.434** | | **11.9698** | **< 0.001** | | | |  |
| **Time** | **2** | | | **88.268** | | | | **44.134** | | **27.1832** | **< 0.001** | | | |  |
| Mesh x Filter | 4 | | | 6.148 | | | | 1.537 | | 0.9467 | 0.440 | | | |  |
| Mesh x Time | 2 | | | 1.899 | | | | 0.950 | | 0.5849 | 0.559 | | | |  |
| Filter x Time | 8 | | | 21.541 | | | | 2.693 | | 1.6584 | 0.116 | | | |  |
| Mesh x Filter x Time | 8 | | | 4.650 | | | | 0.581 | | 0.3580 | 0.941 | | | |  |
| Residuals | 120 | | | 194.828 | | | | 1.624 | |  |  | | | |  |
| **Oak (*Quecus robur* L.)** | | | | | | | | | | | | | |  |  |
| **Factors** | **d.f.** | | | | **SS** | | **MS** | | **F** | | | | ***p*** | |  |
| Mesh | 1 | | | | 0.023 | | 0.0235 | | 0.0180 | | | | 0.894 | |  |
| **Filter** | **4** | | | | **80.622** | | **20.1556** | | **15.4383** | | | | **< 0.001** | |  |
| **Time** | **2** | | | | **51.577** | | **25.7885** | | **19.7529** | | | | **< 0.001** | |  |
| **Mesh x Filter** | **4** | | | | **28.015** | | **7.0038** | | **5.3646** | | | | **< 0.001** | |  |
| Mesh x Time | 2 | | | | 0.982 | | 0.4909 | | 0.3760 | | | | 0.687 | |  |
| Filter x Time | 8 | | | | 20.432 | | 2.5539 | | 1.9562 | | | | 0.058 | |  |
| Mesh x Filter x Time | 8 | | | | 5.852 | | 0.7315 | | 0.5603 | | | | 0.808 | |  |
| Residuals | 120 | | | | 156.667 | | 1.3056 | |  | | | |  | |  |
| **Beech (*Fagus sylvatica* L.)** | | | | | | | | | | | | | | | |
| **Factors** | | **d.f.** | **SS** | | | **MS** | | | | **F** | | ***p*** | | | |
| **Mesh** | | **1** | **43.56** | | | **43.556** | | | | **5.8811** | | **0.017** | | | |
| **Filter** | | **4** | **288.45** | | | **72.113** | | | | **9.7371** | | **< 0.001** | | | |
| **Time** | | **2** | **359.53** | | | **179.766** | | | | **24.2731** | | **< 0.001** | | | |
| Mesh x Filter | | 4 | 8.60 | | | 2.151 | | | | 0.2905 | | 0.884 | | | |
| Mesh x Time | | 2 | 8.88 | | | 4.441 | | | | 0.5997 | | 0.551 | | | |
| **Filter x Time** | | **8** | **142.10** | | | **17.763** | | | | **2.3984** | | **0.020** | | | |
| Mesh x Filter x Time | | 8 | 36.76 | | | 4.595 | | | | 0.6204 | | 0.759 | | | |
| Residuals | | 120 | 888.72 | | | 7.406 | | | |  | |  | | | |

Table S11 ANOVAs' results of three categorical factors (Mesh: mesh size with 2 levels, Filter with 5 levels and Time with 3 levels) and their interactions on a single dependent variable: C:N for the three species. Degrees of freedom (d.f.), sum of squares (SS), mean square (MS), F statistic (F) and p-value (p) are presented. Significant terms are shown in bold. Since dropping non-significant terms didn’t change the model, we decided to keep them.

**
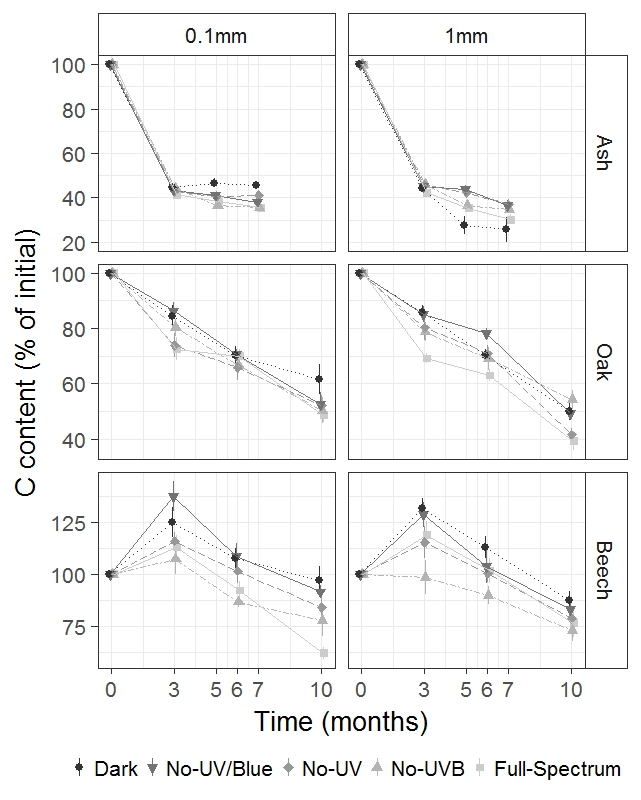
**

Figure S7 Carbon content in percentage of initial weight for each species litter, mesh size and filter treatment. Means ± SE are shown (n = 5)

**
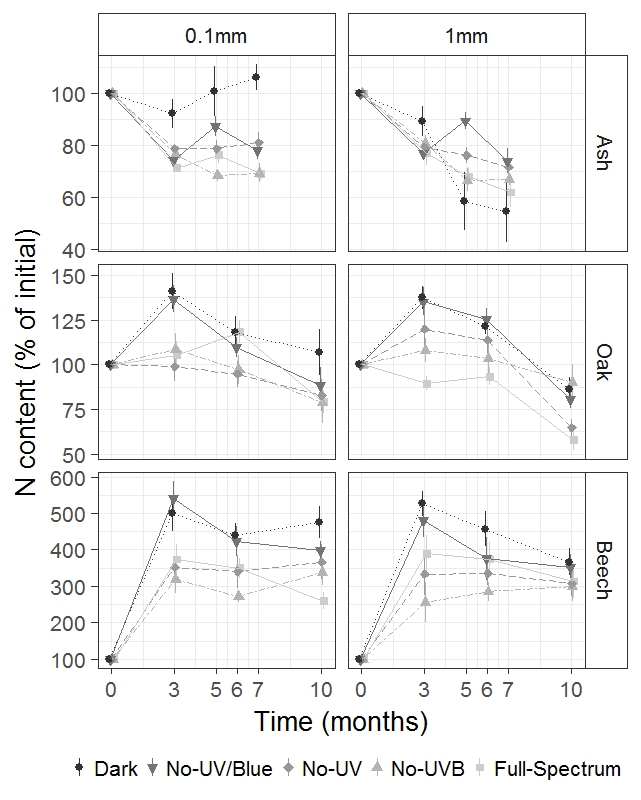
**

Figure S8 Nitrogen content in percentage of initial weight for each species litter, mesh size and filter treatment. Means ± SE are shown (n = 5)


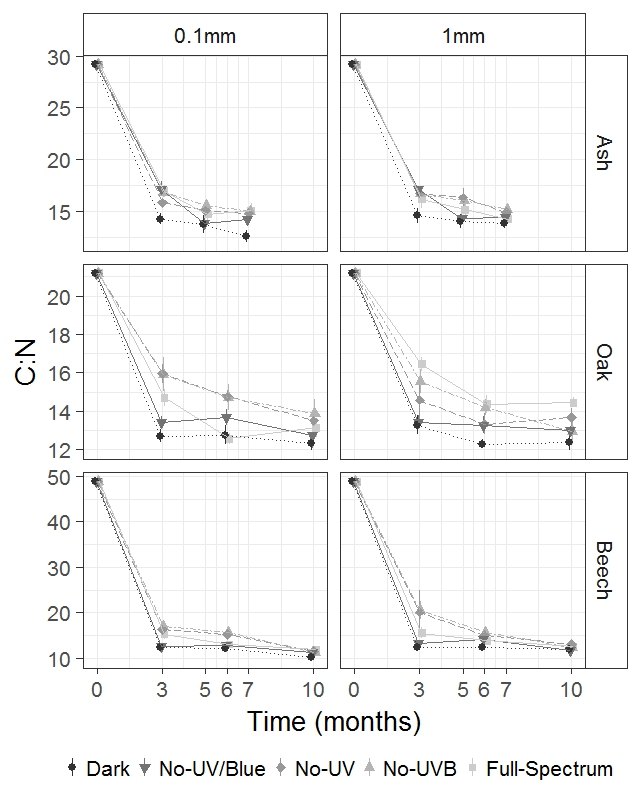


Figure S9 C:N ratio for each species litter, mesh size and filter treatment. Means ± SE are shown (n = 5).


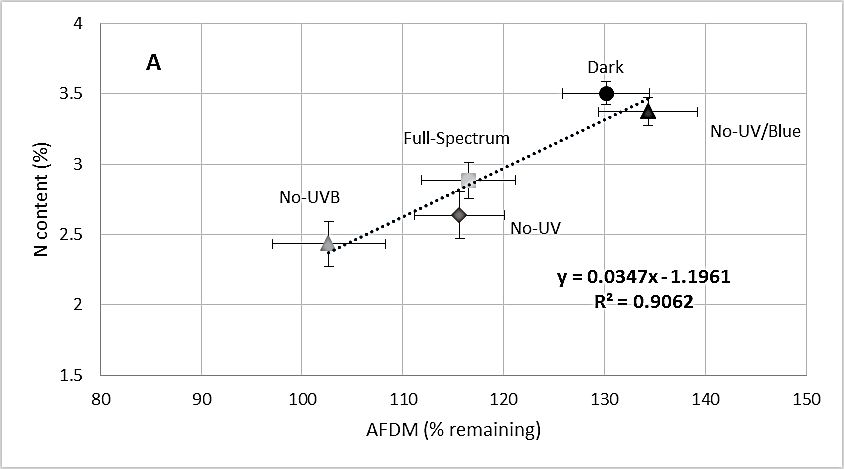


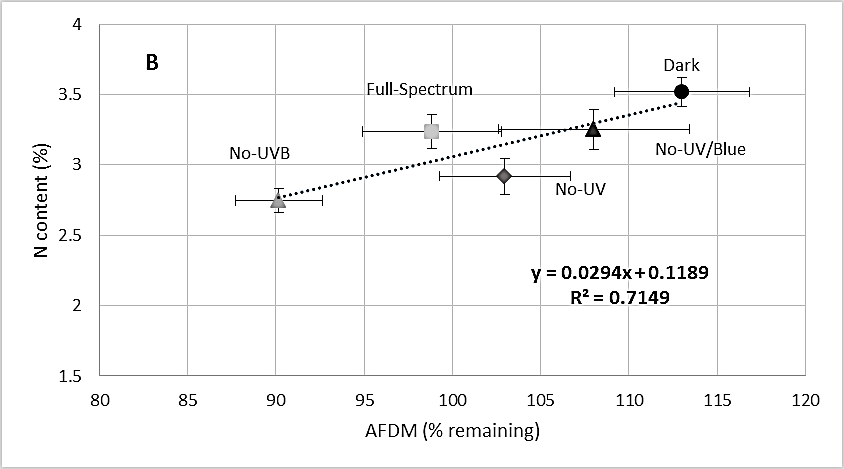


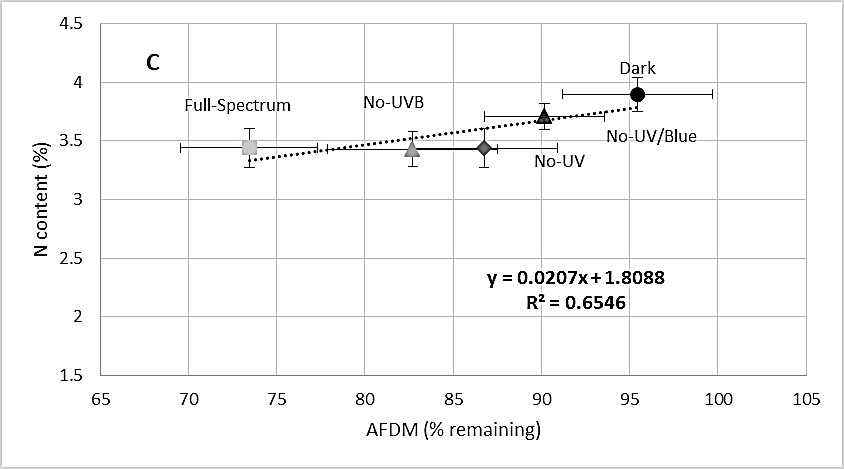


Figure S10 Scatterplot showing the linear regression and the coefficient of determination (R2) between remaining mass and N content in beech litter at the three collection dates, (A) 3 months, (B) 6 months and (C) 10 months after deployment. Means and standard errors are shown for each treatment (n=10).

| **Collection time**  **(months)** | **Filter treatment**  **/unfiltered** | **UV-B** | **UV-A** | **Blue light** | **PAR** |
| --- | --- | --- | --- | --- | --- |
|  |  | **(mmol m-2 day-1)** | **(mol m-2 day-1)** | **(mol m-2 day-1)** | **(mol m-2 day-1)** |
| 3 | Dark | -5.1668 | 0.0282 | 0.4469 | 2.9607 |
|  | No-UV/blue | -5.5027 | 0.1222 | 5.6734 | 1324.7577 |
|  | No-UV | 28.4433 | 16.0595 | 408.0265 | 2010.1689 |
|  | No-UVB | 22.8178 | 34.2465 | 413.7614 | 2024.6098 |
|  | Full-Spectrum | 820.0680 | 40.6450 | 411.3940 | 2023.8637 |
|  | ***Unfiltered*** | ***924.3216*** | ***43.9426*** | ***429.5084*** | ***2094.3745*** |
| 5 | Dark | -8.6741 | 0.0477 | 0.7508 | 5.0862 |
|  | No-UV/blue | -9.2380 | 0.2068 | 9.5318 | 2275.8206 |
|  | No-UV | 47.7509 | 27.1667 | 685.5224 | 3453.2986 |
|  | No-UVB | 38.3067 | 57.9323 | 695.1576 | 3478.1068 |
|  | Full-Spectrum | 1376.7378 | 68.7562 | 691.1802 | 3476.8251 |
|  | ***Unfiltered*** | ***1551.7595*** | ***74.3347*** | ***721.6139*** | ***3597.9567*** |
| 6 | Dark | -8.9349 | 0.0491 | 0.7659 | 5.2476 |
|  | No-UV/blue | -9.5157 | 0.2131 | 9.7237 | 2348.0531 |
|  | No-UV | 49.1863 | 27.9985 | 699.3250 | 3562.9032 |
|  | No-UVB | 39.4582 | 59.7062 | 709.1542 | 3588.4988 |
|  | Full-Spectrum | 1418.1227 | 70.8615 | 705.0966 | 3587.1764 |
|  | ***Unfiltered*** | ***1598.4056*** | ***76.6108*** | ***736.1431*** | ***3712.1525*** |
| 7 | Dark | -9.2452 | 0.0508 | 0.7830 | 5.4303 |
|  | No-UV/blue | -9.8462 | 0.2205 | 9.9409 | 2429.7929 |
|  | No-UV | 50.8945 | 28.9716 | 714.9442 | 3686.9341 |
|  | No-UVB | 40.8285 | 61.7813 | 724.9929 | 3713.4207 |
|  | Full-Spectrum | 1467.3717 | 73.3244 | 720.8448 | 3712.0522 |
|  | ***Unfiltered*** | ***1653.9156*** | ***79.2734*** | ***752.5847*** | ***3841.3791*** |
| 10 | Dark | -10.0205 | 0.0551 | 0.8250 | 5.8786 |
|  | No-UV/blue | -10.6719 | 0.2389 | 10.4739 | 2630.3926 |
|  | No-UV | 55.1626 | 31.3887 | 753.2758 | 3991.3213 |
|  | No-UVB | 44.2526 | 66.9357 | 763.8633 | 4019.9946 |
|  | Full-Spectrum | 1590.4300 | 79.4417 | 759.4927 | 4018.5131 |
|  | ***Unfiltered*** | ***1792.6180*** | ***85.8871*** | ***792.9344*** | ***4158.5170*** |

Table S12 Estimated cumulated doses of UV-B and UV-A radiation, blue light and total PAR received by the litter under different filter treatments at each collection time, compared with unfiltered conditions. Estimates obtained by applying transmittance parameter of the filters measured using an array spectroradiometer (Maya2000 Pro Ocean Optics, Dunedin, FL, USA; D7-H-SMA cosine diffuser, Bentham Instruments Ltd, Reading, UK) that had been calibrated for measurements in the solar UV and visible radiation within the previous 12 months.

| **Variable** | **Factors** | **d.f.** | **SS** | **MS** | **F** | ***p*** |
| --- | --- | --- | --- | --- | --- | --- |
| ***Temperature*** | Mesh | 1 | 6.5 | 6.46 | 0.9663 | 0.325 |
|  | **Filter** | **3** | **84.5** | **28.17** | **4.2137** | **0.006** |
|  | Mesh x Filter | 3 | 6.3 | 2.11 | 0.3158 | 0.814 |
|  | Residuals | 1144 | 7646.8 | 6.68 |  |  |
| ***Moisture*** | **Mesh** | **1** | **198** | **197.55** | **5.9319** | **0.02** |
|  | **Filter** | **3** | **1476** | **491.99** | **14.7729** | **< 0.001** |
|  | Mesh x Filter | 3 | 252 | 84.16 | 2.5269 | 0.06 |
|  | Residuals | 1144 | 38100 | 33.30 |  |  |

Table S. 13 ANOVAs' results of two categorical factors (Mesh: mesh size with 2 levels, Filter with 5 levels) and their interactions on a single dependent variable: temperature (above) and moisture (below) measured inside the different treatments during the decomposition study. Degrees of freedom (d.f.), sum of squares (SS), mean square (MS), F statistic (F) and p-value (p) are presented.

| **Temperature** | | | | |
| --- | --- | --- | --- | --- |
| **Filter** | **Estimate** | **SE** | **t-value** | **P value** |
| Dark - No-UV/Blue | 0.149266 | 0.21545 | 0.228666 | 0.819169 |
| **Dark - No-UVB** | **-0.50544** | **0.21545** | **-2.81014** | **0.025182** |
| Dark - Full-Spectrum | -0.22852 | 0.21545 | -1.06068 | 0.578119 |
| **No-UV/Blue - No-UVB** | **-0.52471** | **0.21545** | **-3.0388** | **0.014575** |
| Full-Spectrum – No-UV/Blue | -0.42619 | 0.21545 | -1.97812 | 0.192618 |
| Full-Spectrum – No-UVB | -0.37692 | 0.21545 | -1.74946 | 0.241441 |
| **Moisture** | | | | |
| **Filter** | **Estimate** | **SE** | **t-value** | **P value** |
| Dark - No-UV/Blue | 0.021253 | 0.480912 | 0.044193 | 0.965 |
| **Dark - No-UVB** | **2.689758** | **0.480912** | **5.593037** | **< 0.001** |
| **Dark - Full-Spectrum** | **1.57651** | **0.480912** | **3.278167** | **0.004** |
| **No-UV/Blue - No-UVB** | **2.668505** | **0.480912** | **5.548844** | **< 0.001** |
| **Full-Spectrum – No-UV/Blue** | **-1.55526** | **0.480912** | **-3.23397** | **0.004** |
| **Full-Spectrum – No-UVB** | **1.113249** | **0.480912** | **2.31487** | **0.042** |
| **Mesh** | **Estimate** | **SE** | **t-value** | **P value** |
| **0.1 mm – 1 mm** | **0.828223** | **0.340056** | **2.435547** | **0.015** |

Table S. 14 Pairwise comparisons for filter treatments and mesh size on temperature and moisture: t- tests, with the Holm’s correction for multiple comparisons, were used to calculate the *P* values. Significant contrasts are shown in bold.
